# A computational method to design broad-spectrum T cell-inducing vaccines applied to *Betacoronaviruses*

**DOI:** 10.1016/j.crmeth.2026.101508

**Published:** 2026-06-29

**Authors:** Phil Palmer, Sofiya Fedosyuk, Srivatsan Parthasarathy, Jonathan Holbrook, Charlotte George, Laura O’Reilly, Lara Wiegand, George William Carnell, Jonathan Luke Heeney, Sneha Vishwanath

**Affiliations:** 1 Lab of Viral Zoonotics, Department of Veterinary Medicine, University of Cambridge, Cambridge CB3 0ES, UK; 2 DIOSynVax Ltd, University of Cambridge, Cambridge CB3 0ES, UK; 3 Institute of Medical Microbiology and Hygiene, University Hospital Regensburg, 93053 Regensburg, Germany; 4 One Virology, School of Veterinary Medicine and Science, University of Nottingham, Sutton Bonington Campus, Loughborough LE12 5RD, UK

**Keywords:** computational vaccine design, T cell vaccines, cross-reactive immunity, *Betacoronaviruses*, SARS-CoV-2, MERS-CoV, HLA diversity, epitope prediction, pandemic preparedness, broad-spectrum vaccines, population coverage, artificial intelligence, immunoinformatics, antigen design, nucleocapsid protein

## Abstract

Antigenically diverse pathogens such as coronaviruses pose substantial global health threats, highlighting the need for broad-spectrum vaccines. Here, we introduce Spectravax, a computational method that designs broad-spectrum vaccines accounting for genetic diversity in both host and pathogen populations. Using Spectravax, we designed a nucleocapsid (N) antigen to elicit cross-reactive immune responses to viruses from the *Sarbecovirus* and *Merbecovirus* subgenera of *Betacoronaviruses*. *In silico* analyses demonstrated superior predicted host and pathogen coverage for Spectravax compared to wild-type sequences and existing computational designs. Experimental validation in mice supported these predictions: Spectravax N elicited robust immune responses to SARS-CoV, SARS-CoV-2, and MERS-CoV—the three coronaviruses responsible for major outbreaks in humans since 2002—while wild-type and existing computational designs elicited limited responses. Furthermore, we identified the MERS-CoV N epitopes responsible for Spectravax’s cross-reactivity, advancing the rational design of broad-spectrum vaccines for pandemic preparedness.

## Highlights

• Spectravax is a computational method for broad-spectrum T cell vaccine antigen design
• We use Spectravax to design an N antigen with broad host and pathogen coverage
• Spectravax N elicits cross-reactive T cell responses to three *Betacoronaviruses*
• Immunodominant MERS-CoV N epitopes underpin cross-reactive immunity

## Motivation

*Betacoronaviruses* pose a sustained pandemic threat, with SARS-CoV, SARS-CoV-2, and MERS-CoV causing major outbreaks in humans since 2002. Broad T cell immunity targeting conserved internal proteins offers a promising route to cross-reactive protection across antigenically diverse variants and subgenera. Achieving such protection requires vaccine antigens that simultaneously account for human immunological diversity and viral sequence diversity, yet these dimensions are typically considered separately in vaccine design. We address this challenge with Spectravax, a framework that integrates peptide-MHC binding predictions with HLA allele frequencies and pathogen sequence conservation to design single-antigen vaccines predicted to provide broad population coverage across multiple *betacoronavirus* subgenera.

## Graphical abstract

**Figure:**
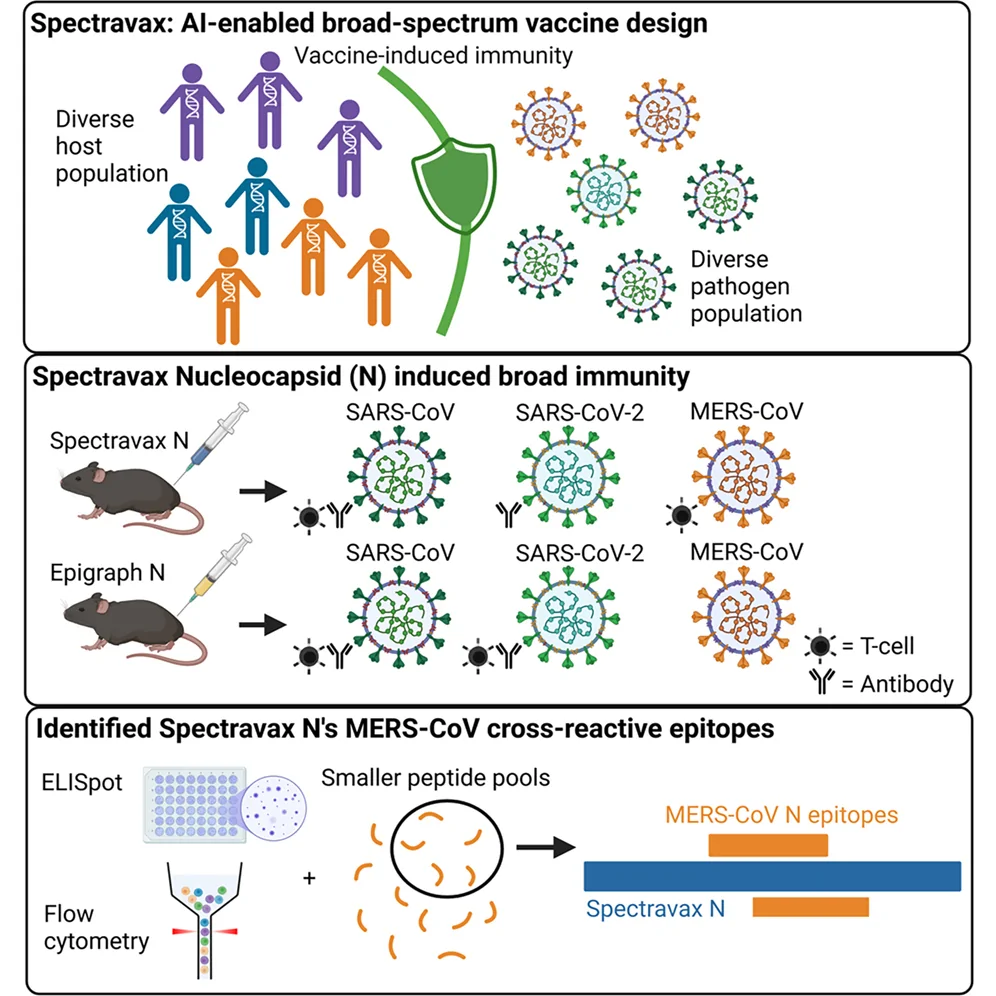

## Teaser

Palmer et al. present Spectravax, a computational method for designing broad-spectrum T cell vaccine antigens that accounts for genetic diversity in both host and pathogen populations. Applied to *Betacoronaviruses*, Spectravax elicited cross-reactive T cell immunity against SARS-CoV, SARS-CoV-2, and MERS-CoV in mice.

## Introduction

Antigenically diverse and rapidly evolving pathogens, such as coronaviruses, pose substantial challenges to global health. Vaccines are among the most cost-effective medical interventions and are estimated to have saved 20 million lives from the severe acute respiratory syndrome coronavirus 2 (SARS-CoV-2) pandemic in 2021 alone.^1^ However, most available vaccines are designed to target a single strain or closely related strains. This limitation reduced their effectiveness against emerging variants, such as Omicron and its sublineages, which evaded immune responses generated by prior infection, vaccination, or both.^2^ Additionally, other *Betacoronaviruses* (*β*-CoVs), including viruses from the *Sarbecovirus* and *Merbecovirus* subgenera, pose risks of zoonotic spillover.^3^ This emphasizes the need for broad-spectrum vaccines that provide protection across multiple strains and variants.

According to recent estimates, a broadly protective coronavirus vaccine could have prevented 40%–65% of COVID-19 deaths during the pandemic’s first year^4^ and could be worth at least $1.5 trillion in social value to the US alone.^5^ Calls for a pan-*β*-CoV vaccine aim to prevent diseases caused by SARS-CoV, SARS-CoV-2, Middle East respiratory syndrome coronavirus (MERS-CoV), and future emergent coronaviruses.^6^ In response, substantial investments have been made toward developing pan-coronavirus vaccines.^7^ However, most pan-*β*-CoV vaccines in development use multivalent approaches, such as nanoparticles displaying multiple antigens from different viruses,^8^^,^^9^ which may face manufacturing and regulatory challenges due to their complexity.^10^

Computational methods offer a generalizable solution for designing single-antigen broad-spectrum vaccines, as they can easily be applied to other pathogens.^11^ These computationally designed antigens can serve as alternatives to, or components of, multivalent vaccines, enhancing their breadth.^12^ Approaches like Epigraph^13^ generate mosaic antigens that maximize coverage of pathogen sequence diversity. Deep-learning-based immunoinformatics tools such as NetMHCpan^14^ predict peptide-major histocompatibility complex (MHC) binding affinities, enabling the selection of epitopes that are more likely to elicit T cell responses. OptiVax^15^ combines these predictions with human leukocyte antigen (HLA) haplotype frequencies to optimize peptide-based vaccine designs for host population coverage. However, few methods integrate pathogen diversity and host immunogenicity to design conserved and immunogenic vaccines,^15^^,^^16^^,^^17^ and none integrate both host and pathogen coverage to maximize the number of individuals protected against the greatest number of pathogen variants.

Here, we focus on predicting T cell epitopes, which is currently more tractable than predicting B cell epitopes due to their simpler, short linear peptide sequences, as opposed to the complex three-dimensional structures critical for B cell epitope recognition.^14^^,^^18^^,^^19^ Focusing on T cell-mediated immunity offers additional benefits. T cell-mediated immunity is crucial for controlling viral infections and provides long-lasting protection.^20^ T cells can recognize conserved internal viral proteins that are less susceptible to mutation, offering broader protection against diverse strains.^19^ In coronaviruses, robust T cell responses have been associated with effective clearance of infections,^21^^,^^22^ milder disease,^23^ reduced transmission,^24^ long-lasting immunity,^25^ and decreased susceptibility to immune escape.^26^ The nucleocapsid (N) protein is highly conserved and immunogenic, and N-specific T cells are correlated with protection against SARS-CoV-2 infection,^27^ making it an ideal candidate for broad-spectrum vaccine design.^28^

In this work, we present Spectravax, a computational framework and method to design single-antigen broad-spectrum vaccines optimized to account for genetic diversity in both pathogen antigens and host MHCs. Spectravax leverages peptide-MHC binding predictions and host HLA allele frequencies to select antigen sequences predicted to be widely recognized by T cells across diverse populations, while using k-mer frequencies to ensure conservation across pathogen strains. We applied this method to design an N antigen (Spectravax N) intended to elicit cross-reactive immune responses to viruses from the *Sarbecovirus* and *Merbecovirus* subgenera. Our analyses show that Spectravax N is predicted to display a wide range of T cell epitopes across a diverse set of HLA alleles, providing robust host population coverage across three different ancestries and 95 countries. Furthermore, Spectravax N has high sequence similarity to N proteins from *Sarbecoviruses* and *Merbecoviruses* and is the only vaccine candidate in our study predicted to induce T cell responses to all target pathogen sequences when compared to wild-type sequences and a vaccine design generated using Epigraph.^13^ We chose to compare our design with Epigraph, as our method uses a similar graph-based algorithm for vaccine design. Experimental validation in a mouse model demonstrated that Spectravax N elicited immune responses against all three tested coronaviruses, while the wild-type and Epigraph antigens induced narrower responses. Finally, using smaller peptide pools from MERS-CoV N for enzyme-linked immunospot (ELISpot) and flow cytometry assays, we were able to identify the specific epitopes responsible for Spectravax N’s MERS-CoV reactivity. Together, these findings highlight Spectravax as a promising approach for the rational design of broad-spectrum vaccines.

## Results

### Target antigen selection

**Figure 1:**
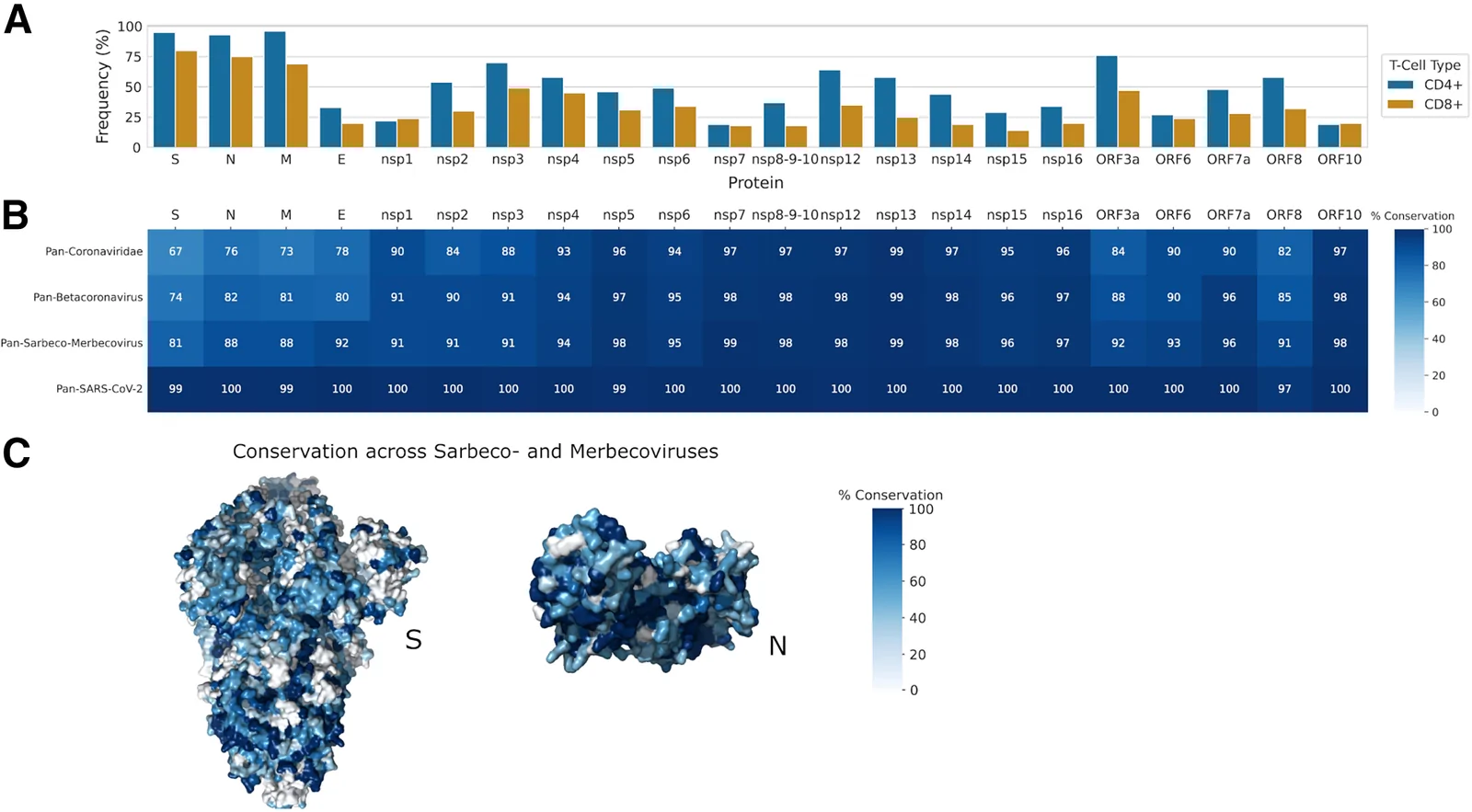
Conservation and T cell immunogenicity of *Coronaviridae* proteins (A) Reported CD4^+^ and CD8^+^ immunogenicity of SARS-CoV-2 proteins in convalescent COVID-19 donors (*n* = 99).^29^ (B) Protein sequence conservation of coronavirus proteins across the *Coronaviridae* (family), *Betacoronavirus* (genus), *Sarbecovirus* and *Merbecovirus* (subgenera), and SARS-CoV-2 (species). For each taxonomic group, up to 100 protein sequences were randomly sampled from complete genome annotations and individual protein records in NCBI GenBank^30^ (accessed 2nd June 2025). Sequences were clustered at 99% identity using CD-HIT^31^ to remove near-duplicates and then aligned using MAFFT.^32^ Conservation scores represent the mean percentage identity to the most common amino acid at each position, excluding gaps. For the nsp8-9-10 complex, conservation scores were averaged across the three individual proteins. (C) Surface representation of the spike (S) ectodomain trimer (PDB: 6VSB)^33^ and nucleocapsid (N) single subunit (PDB: 8FG2)^34^ of SARS-CoV-2. The amino acids are color coded according to the sequence conservation across the *Sarbecovirus* and *Merbecovirus* subgenera. The color bar represents the percentage of conservation. The image was generated and rendered using Pymol Open-Source v.2.5.0.^35^

We selected the N protein as the target antigen based on its high T cell immunogenicity and sequence conservation across the *Sarbecovirus* and *Merbecovirus* subgenera (Figure 1).

The N protein has the second-highest total T cell immunogenicity among SARS-CoV-2 proteins, only slightly below the spike (S) protein (Figure 1A). Specifically, 93% and 75% of individuals responded to the N protein for CD4^+^ and CD8^+^ T cells, respectively.^29^

However, the N protein is more conserved than the S protein, with a sequence conservation of 88% across the *Sarbecovirus* and *Merbecovirus* subgenera, compared to 81% for the S protein (Figure 1B). Higher sequence conservation is observed across the surface of the N protein compared to that of the S protein (Figure 1C).

### Designing a single-antigen vaccine to target *Sarbecoviruses* and *Merbecoviruses*

We applied Spectravax to design an N protein vaccine antigen. We formulate the objective of Spectravax as designing a protein sequence that protects as many individuals from the target host population as possible against as many pathogens from the target pathogen population as possible. Here, the target host population was the global human population, and the target pathogen population consisted of *β*-CoVs from the *Sarbecovirus* and *Merbecovirus* subgenera.

**Figure 2:**
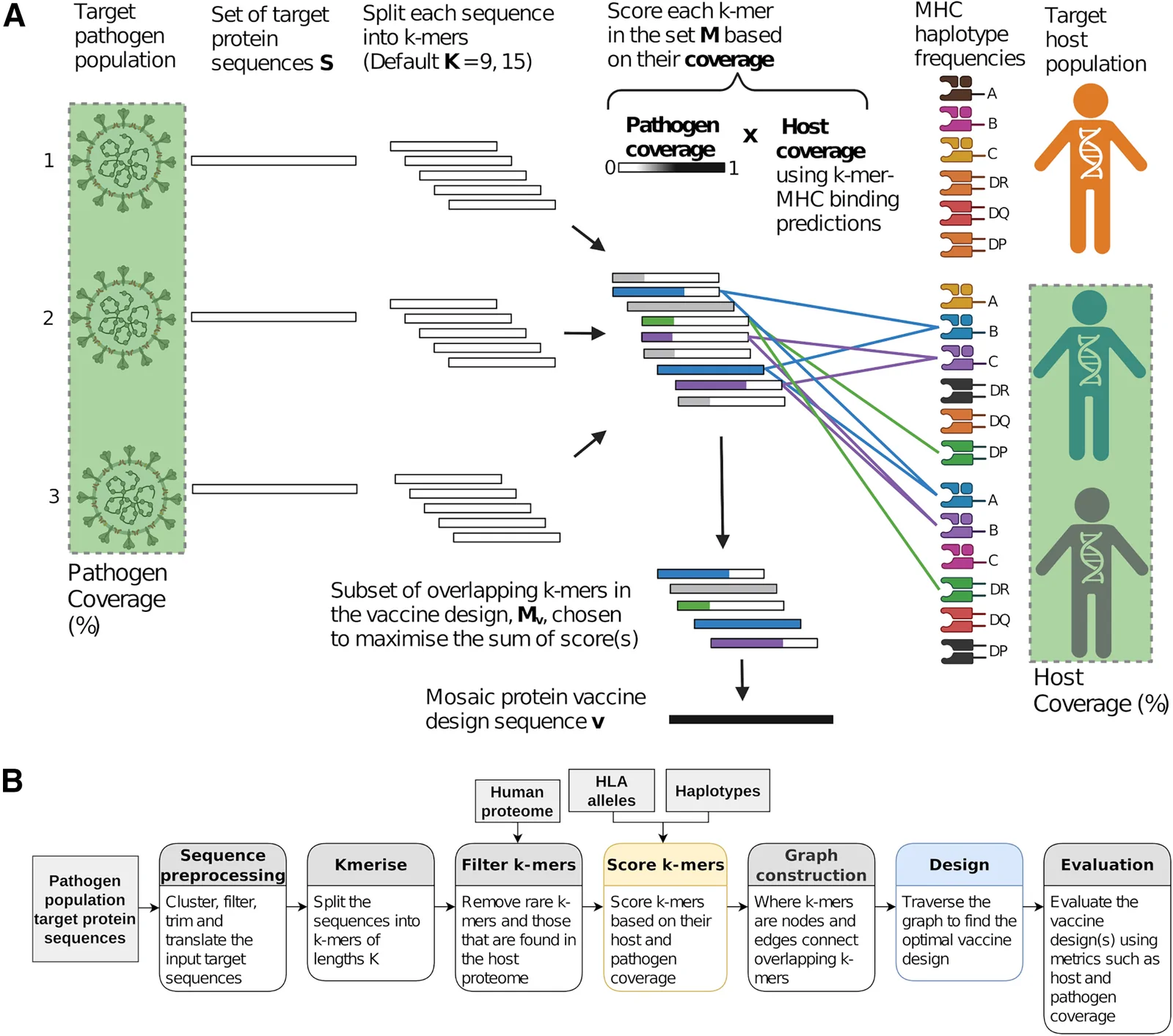
The Spectravax framework (A) Overview of the Spectravax vaccine design aiming to maximize coverage of host and pathogen populations. Pathogen coverage is shown as the fraction of pathogen variants (green highlighted viruses) containing peptides in the vaccine design. Host coverage is represented by the fraction of individuals (green highlighted figures) predicted to present vaccine-derived peptides via their HLA alleles. (B) Spectravax computational workflow: sequence preprocessing, k-mer generation, filtering, scoring, graph construction, vaccine design, and evaluation.

Spectravax fragments target pathogen protein sequences into short subsequences called k-mers, mimicking antigen processing by the immune system (Figure 2A). These k-mers are then filtered and scored using two coverage metrics.

(1) Pathogen coverage: the fraction of target pathogen protein sequences that contain a given k-mer. This metric measures how widely a k-mer is conserved across the pathogen population, indicating its potential to protect against multiple variants.
(2) Host coverage: the fraction of individuals in the target host population predicted to present at least *n* peptides from a given set of k-mers (default *n* = 1). This metric assesses the likelihood that individuals can present vaccine-derived peptides on their MHC molecules, determined by peptide-MHC binding predictions combined with HLA allele frequencies.

For each k-mer, the host and pathogen coverage scores are multiplied to obtain a total coverage score. The Epigraph algorithm^13^ was reimplemented in Python using the NetworkX library^36^ and used to select the optimal overlapping subset of k-mers to generate the vaccine design (Figure S1).

We chose to design a contiguous protein sequence vaccine rather than a set of peptides or a linker-protein vaccine because, by leveraging overlaps, mosaic vaccines can include more epitopes for the same number of amino acids,^37^^,^^38^ enhancing immunogenicity and avoiding artificial junctions.^39^ The Spectravax workflow (Figure 2B) is further detailed in the STAR Methods section.

### Overview of the Spectravax N vaccine design

**Figure 3:**
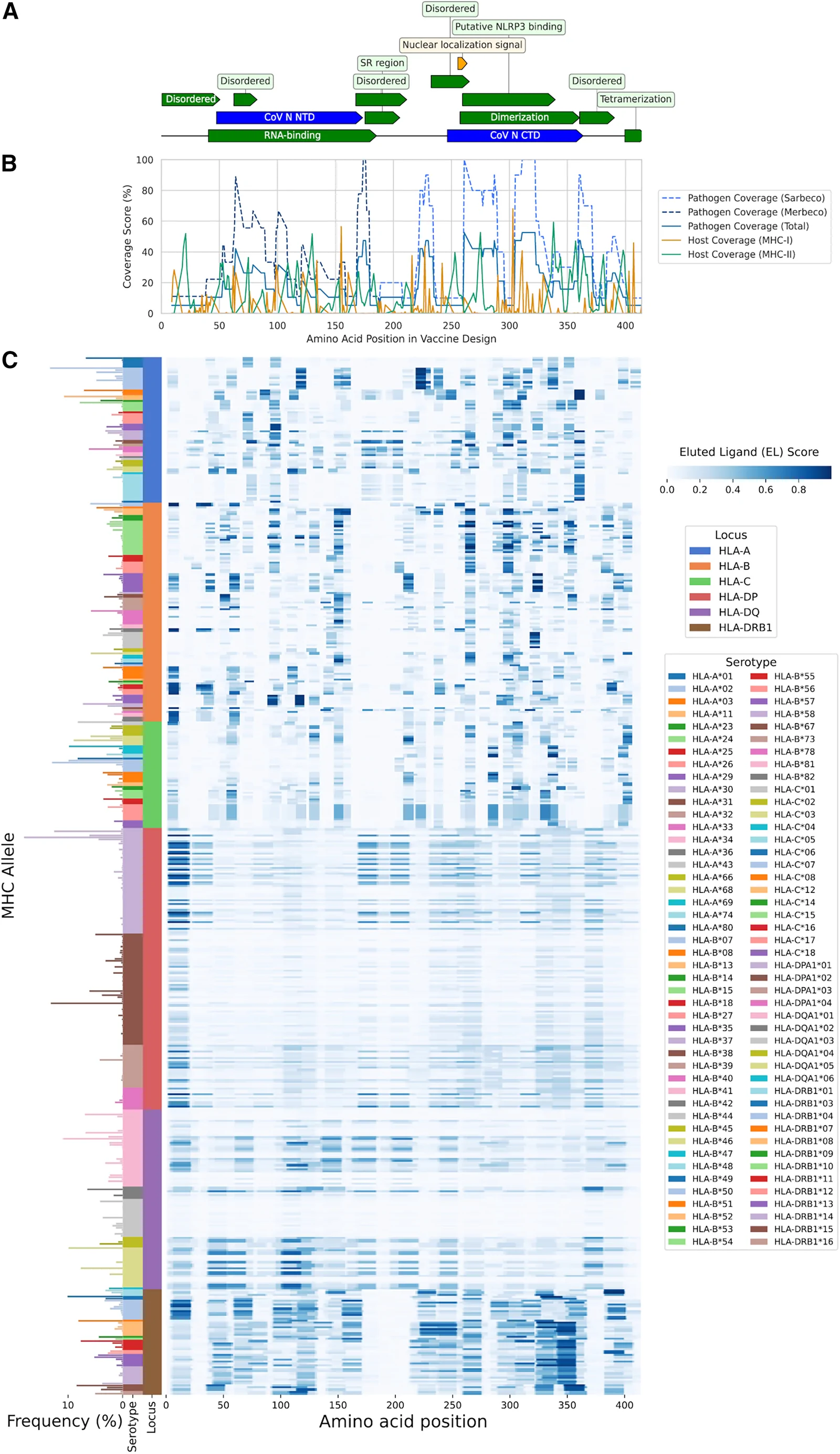
Overview of Spectravax N by amino acid position (A) SARS-CoV-2 N protein (UniProt ID: P0DTC9) annotated with domains (blue), regions (green), and motifs (orange). (B) The Spectravax N antigen, showing k-mer scores for pathogen coverage (blue), *Sarbecovirus* only (light blue), *Merbecovirus* only (dark blue), MHC-I host coverage (orange), and MHC-II host coverage (green). (C) Peptide-MHC presentation heatmap for Spectravax N. The *x* axis represents amino acid position; the *y* axis represents HLA alleles, grouped by locus and serotype. The inverted *x* axis shows allele frequencies in the global population. The color intensity indicates the eluted ligand (EL) score, reflecting the likelihood of peptide presentation by MHC molecules. See also Figure S2.

The Spectravax N antigen is a protein sequence of 414 amino acids (Figure 3). 3D structural modeling of the Spectravax N antigen using AlphaFold2^40^ confirmed that the structured N- and C-terminal domains adopt similar folds to the wild-type protein (Figure S2).

The N-terminal domain (NTD) of the antigen design consists entirely of *Merbecovirus* 9-mers, while the C-terminal domain (CTD) contains only *Sarbecovirus* 9-mers, with a transition at amino acid position 188 (Figures 3A and 3B). This separation in the antigen design reflects the distinct nature of the subgenera, which causes each subgenus to have its own region in the antigen design to maximize the coverage of both.

The pathogen coverage remains high across the designed protein, reflecting its high conservation—all k-mers have non-zero pathogen coverage as they are present in at least one target sequence. In contrast, the host population coverage scores are lower and variable, as only a subset of k-mers are predicted to be presented by MHC class I (MHC-I) or MHC class II (MHC-II) molecules (Figure 3B).

The antigen contains numerous k-mers predicted to be presented by a wide range of MHC alleles (MHC-I and MHC-II), essential for effective host coverage (Figure 3C).

The heatmaps reveal “hotspots” where certain regions of the protein are more likely to be presented by MHC molecules. For instance, amino acid positions 5–11 and 262–270 show higher predicted presentation (mean eluted ligand [EL] score over 0.2), while regions such as 185–196 and 408–414 show lower presentation (mean EL score below 0.05).

Differences between MHC-I and MHC-II loci are evident in the peptide-MHC heatmaps. MHC-I loci (HLA-A, HLA-B, and HLA-C) display more distinct and punctuated patterns, with pronounced variation in EL scores between adjacent k-mers. In contrast, MHC-II loci (HLA-DP, HLA-DQ, and HLA-DR) show smoother patterns due to their broader peptide-binding specificity.

Notably, the HLA-DRB1 locus has the highest EL scores, with a mean of 0.23 across all k-mers and alleles—more than twice that of other loci (post hoc Dunn test, adjusted *p* values <0.001). This may be due to biological factors or biases in the NetMHCIIpan training dataset, as HLA-DRB1 alleles are overrepresented in the training dataset.^14^

Alleles sharing the same serotype exhibit similar peptide presentation patterns due to shared peptide-binding motifs. High predicted presentation scores are observed for serotypes like HLA-DRB1∗04, ∗09, and ∗10, while others like HLA-DQA1∗03 show the lowest scores.

### *In silico* analyses of Spectravax N

We conducted computational analyses to evaluate the predicted coverage of Spectravax N across both host and pathogen populations and to compare it with wild-type sequences and an existing computational design.

**Figure 4:**
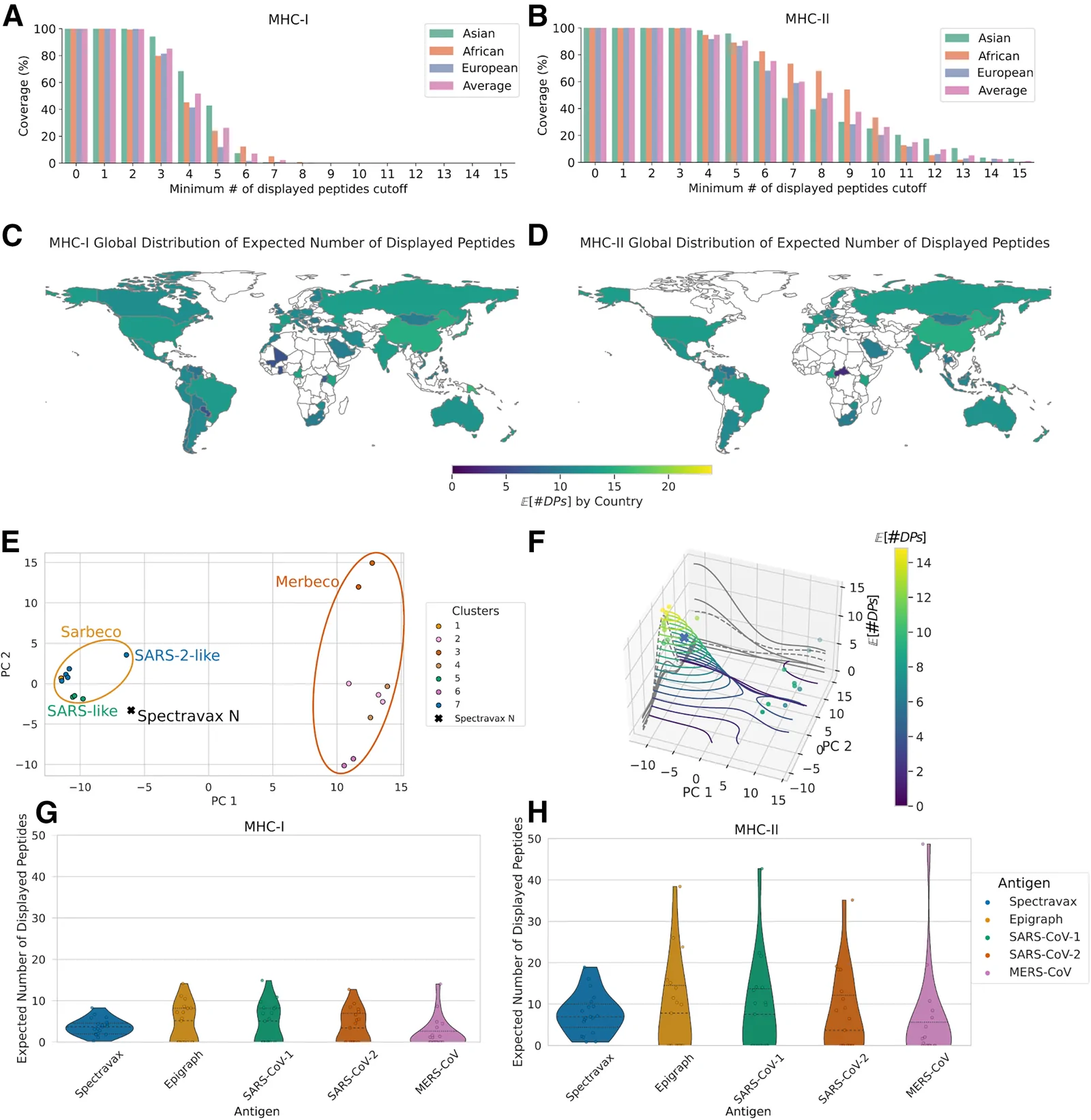
*In silico* analyses of Spectravax N antigen coverage (A and B) Percentage of Asian (green), African (orange), European (blue) individuals, and the combined population (pink) predicted to present at least *n* vaccine-derived peptides for (A) MHC-I and (B) MHC-II. (C and D) Expected number of displayed peptides E[#DPs] across 95 countries for (C) MHC-I and (D) MHC-II. Countries missing data from the Allele Frequency Net Database (AFND) are colored white. (E) Principal-component analysis (PCA) of the N protein sequences from the target pathogens and the Spectravax N antigen in 2D sequence space. Each point represents a target pathogen sequence, colored by automatically assigned clusters, with the Spectravax N design indicated with a black cross. (F) Contour plot of the protein fitness landscape showing the E[#DPs] for each pathogen sequence in the PCA space. (G and H) Violin plots of the coverage, showing the E[#DPs] for each antigen against the target sequences for (G) MHC-I and (H) MHC-II. Colors represent different antigens: Spectravax (blue), Epigraph (yellow), SARS-CoV (green), SARS-CoV-2 (orange), and MERS-CoV (pink). See also Table S1.

Spectravax N provides high predicted coverage across diverse human populations (Figures 4A–4D). To quantify this coverage, we use the expected number of displayed peptides, denoted as E[#DPs], representing the average number of vaccine-derived peptides predicted to be presented by an individual’s HLA molecules.

Firstly, the percentage of individuals with Asian, African, and European ancestries predicted to present at least *n* vaccine-derived peptides for MHC-I and MHC-II was calculated (Figures 4A and 4B). The coverage is consistent across ancestries, with Asian and African populations slightly more likely to present an additional peptide compared to the European population for both MHC classes. Notably, E[#DPs] is consistently higher for MHC-II than for MHC-I across all ancestries. Almost the entire population is predicted to present at least two peptides for MHC-I and at least three peptides for MHC-II, suggesting a robust potential immune response.

Secondly, to assess global coverage, we evaluated E[#DPs] using an independent dataset of allele frequencies from the Allele Frequency Net Database (AFND)^41^ across 95 countries (Figures 4C and 4D). Despite missing data for some countries, particularly in Africa, the strong predicted responses in highly populous countries like China and India contribute substantially to a high global E[#DPs] of 13.2 when weighted by population size. The rankings of E[#DPs] across ancestries were found to be consistent across datasets, reinforcing the robustness of our findings (Table S1).

Thirdly, we evaluated the Spectravax N antigen’s predicted coverage across the target pathogen population, consisting of *β*-CoVs from the *Sarbecovirus* and *Merbecovirus* subgenera, finding that the vaccine design is representative of both subgenera (Figures 4E and 4F). This result is further confirmed by the phylogenetic tree and sequence identity matrix (Figure S3).

To visualize the antigen’s placement within the pathogen sequence space, we performed principal-component analysis (PCA) on the aligned N protein target sequences. When the distribution of pathogen sequences is presented in 2D space, with each point representing a sequence colored by automatically assigned clusters, Spectravax N is positioned between the *Sarbecovirus* and *Merbecovirus* clusters (Figure 4E).

To further assess the pathogen coverage of Spectravax N and the target sequences, we calculated the expected number of displayed peptides (E[#DPs]) for each sequence. Figure 4F presents a contour plot mapping the E[#DPs] values onto the 2D sequence space. In this plot, the first two dimensions correspond to the principal components from the PCA, representing sequence similarity, while the contours and color indicate the E[#DPs] values, analogous to a protein fitness landscape. Spectravax N is located near, but not at, the peak of the fitness landscape due to the clade adjustment process (Figure S4), which balances representation from both subgenera.

Finally, we compared Spectravax N to wild-type N sequences from SARS-CoV, SARS-CoV-2, MERS-CoV, and an antigen designed using the Epigraph method for the same initial set of target sequences (Figures 4G and 4H). The Spectravax antigen shows superior breadth, being the only antigen expected to display a non-zero number of peptides against all target sequences. While the Epigraph design and SARS-CoV have a higher E[#DPs] than the Spectravax design, they fail to cover *Merbecoviruses* effectively. Spectravax N, therefore, offers a balanced approach, ensuring coverage across the diverse *β*-CoV population.

### Immunogenicity studies in mice

**Figure 5:**
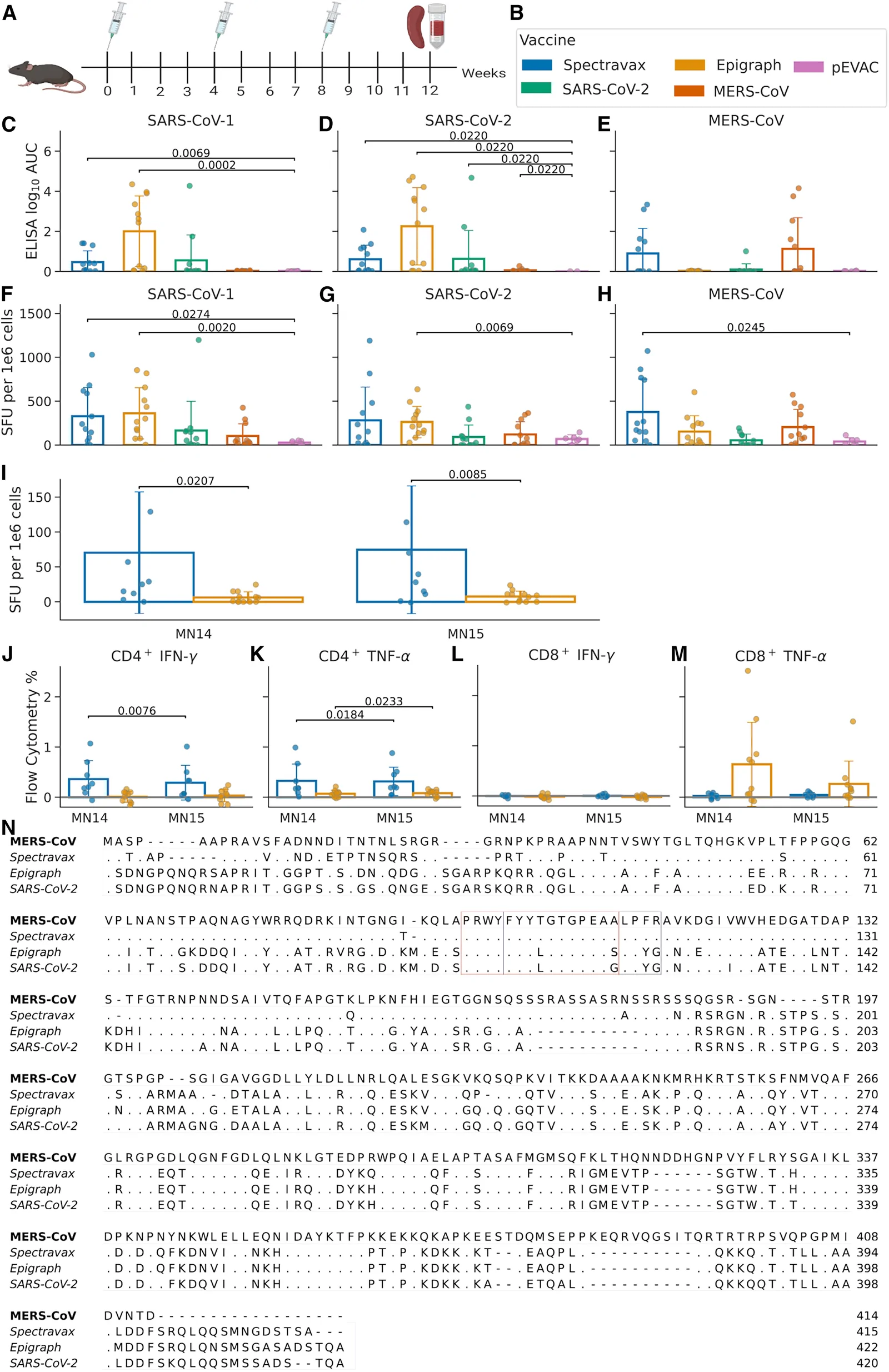
*In vivo* immunogenicity study in the mouse model (A) Immunization schedule. (B) Immunization groups and their color codes: Spectravax (blue), Epigraph (yellow), SARS-CoV-2 (green), MERS-CoV (orange), and negative control (pink). (C–E) ELISA results showing antibody responses against wild-type (C) SARS-CoV, (D) SARS-CoV-2, and (E) MERS-CoV N antigens (Spectravax, Epigraph, SARS-CoV-2, and MERS-CoV antigen groups: *n* = 12 mice each; pEVAC negative control: *n* = 6 mice). (F–H) ELISpot results showing T cell responses measured by IFN-γ production upon stimulation with N peptide pools from (F) SARS-CoV, (G) SARS-CoV-2, and (H) MERS-CoV (same group sizes as in C–E). (I) ELISpot stimulation with MERS-CoV nucleocapsid (MN) peptides MN14 and MN15 only (Spectravax: *n* = 10, Epigraph: *n* = 12). (J–M) Flow cytometry analysis of the following subpopulations of splenocytes: (J) CD4^+^ IFN-γ+, (K) CD4^+^ TNF-α+, (L) CD8^+^ IFN-γ+, and (M) CD8^+^ TNF-α+ in response to MN14 and M15 peptide stimulation (Spectravax *n* = 8, Epigraph *n* = 10). (N) Multiple sequence alignment of the antigens generated using ClustalO.^43^ The regions corresponding to MN14 and MN15 are boxed in red and blue. For (C–H), statistical significance was determined using a two-sided Mann-Whitney U test comparing the Spectravax and Epigraph groups to the negative control. *p* values are shown only for significant differences (*p* < 0.05). For (I–M), *p* values are shown only for significant differences (*p* < 0.05). See also Figure S5 and Table S2.

To test our computational predictions, we experimentally assessed the breadth of the Spectravax N antigen compared to wild-type antigens and an Epigraph-designed antigen using the C57BL/6J mouse model (Figure 5A). Mice were immunized with pEVAC^42^ plasmid DNA encoding one of the antigens (Spectravax N, Epigraph N, SARS-CoV-2 N, or MERS-CoV N) or no antigen (negative control) (Figure 5B). Mice were immunized three times at 4-week intervals, and the study was terminated at week 12, with subsequent collection of sera and splenocytes from all the mice groups (Figure 5A) to characterize the antibody and T cell responses, respectively. Mice sera were evaluated for IgG antibody-binding responses using antigen-specific ELISA (Figures 5C–5E), and mice splenocytes were evaluated for T cell response using IFN-γ ELISpot assays and flow cytometry (Figure 5F–5M).

Spectravax N induced antibody responses against SARS-CoV, SARS-CoV-2, and MERS-CoV with antibody titers comparable to those of homologous wild-type controls. Epigraph N induced robust and stronger antibody responses against the *Sarbecoviruses* but negligible humoral responses against MERS-CoV, while SARS-CoV-2 and MERS-CoV wild-type antigens generated antibodies only against homologous antigens (Figures 5C–5E). As up to 50% of the mice in every immunized group were non-responders, statistical significance could not be established reliably. The antibody responses elicited in responding mice from the Spectravax and Epigraph groups suggest that some surface-exposed epitopes are preserved in both designs, and the Spectravax design has retained some MERS-CoV B cell epitopes that are not present in Epigraph.

T cell responses were initially assessed using ELISpot assays measuring IFN-γ production upon stimulation with peptide pools derived from SARS-CoV, SARS-CoV-2, and MERS-CoV N antigens (Figures 5F–5H). Spectravax N induced significantly higher T cell responses against SARS-CoV and MERS-CoV compared to the negative control (*p* < 0.05), while Epigraph N induced significant T cell responses against the *Sarbecovirus* antigens (*p* < 0.01) but not against MERS-CoV N.

To identify the peptide(s) that led to the differential response to MERS-CoV by the Spectravax and Epigraph antigens, we divided the peptide pools of wild-type MERS-CoV N into smaller peptide pools, with each peptide pool consisting of five to eight peptides. Two mice from each of the Epigraph- and Spectravax-vaccinated mice groups were selected for the initial ELISpot-based screening (Figure S5A). Only the peptide pool MN11-15 elicited T cell responses for both antigens, with Spectravax N eliciting a stronger response compared to Epigraph N. Further, the splenocytes from the two same mice were tested for responses against individual peptides constituting the MN11-MN15 pool (Figure S5B). Responses were observed only for peptides MN14 and MN15, which were computationally predicted to have higher presentation scores (Table S2).

Mice immunized with either Spectravax or Epigraph antigens were then stimulated with the two identified peptides (Figure 5I). Significantly higher T cell responses to peptides MN14 and MN15 were observed in Spectravax-immunized animals compared to Epigraph-immunized animals (*p* < 0.05).

To further delineate which arm of T cell immunity is stimulated by peptides MN14 and MN15, flow cytometry experiments were performed for mice groups immunized with either Spectravax or Epigraph antigens (Figures 5J–5M). CD4^+^-biased responses were observed for Spectravax N, while CD8^+^-biased responses were observed for Epigraph N.

Detailed analyses of peptides MN14 and MN15 show that they have the same core region: “YTGTGPEAA.” These peptides have predicted EL scores of 0.5 and 0.61, respectively, for the mice MHC-II allele H2-IAb and are exact matches between MERS-CoV and Spectravax N, while Epigraph and SARS-CoV-2 antigens have mismatches at two positions in the core region and four positions over the length of the region corresponding to the peptides (Figure 5N). Furthermore, these epitopes may be important for the target human population, as MN14 and MN15 have an expected MHC-II host coverage (STAR Methods) of 30.8% and 10.3%, respectively.

These results demonstrate that even small differences in epitope sequences can substantially impact T cell responses. This emphasizes the importance of computational methods that can enrich antigen sequences with optimal T cell epitopes, particularly for generating broad-spectrum vaccines capable of protecting against multiple target pathogens.

Computational predictions made prior to experimental validation showed a strong correlation with the experimental results, particularly for the ELISA responses: MHC-I *R*_*S*_ = 0.86 and MHC-II *R*_*S*_ = 0.89 (Figure S5C). This correlation suggests that computational predictions can effectively guide vaccine design before time- and resource-intensive experimental validation.

### Generalizability and refinements

**Figure 6:**
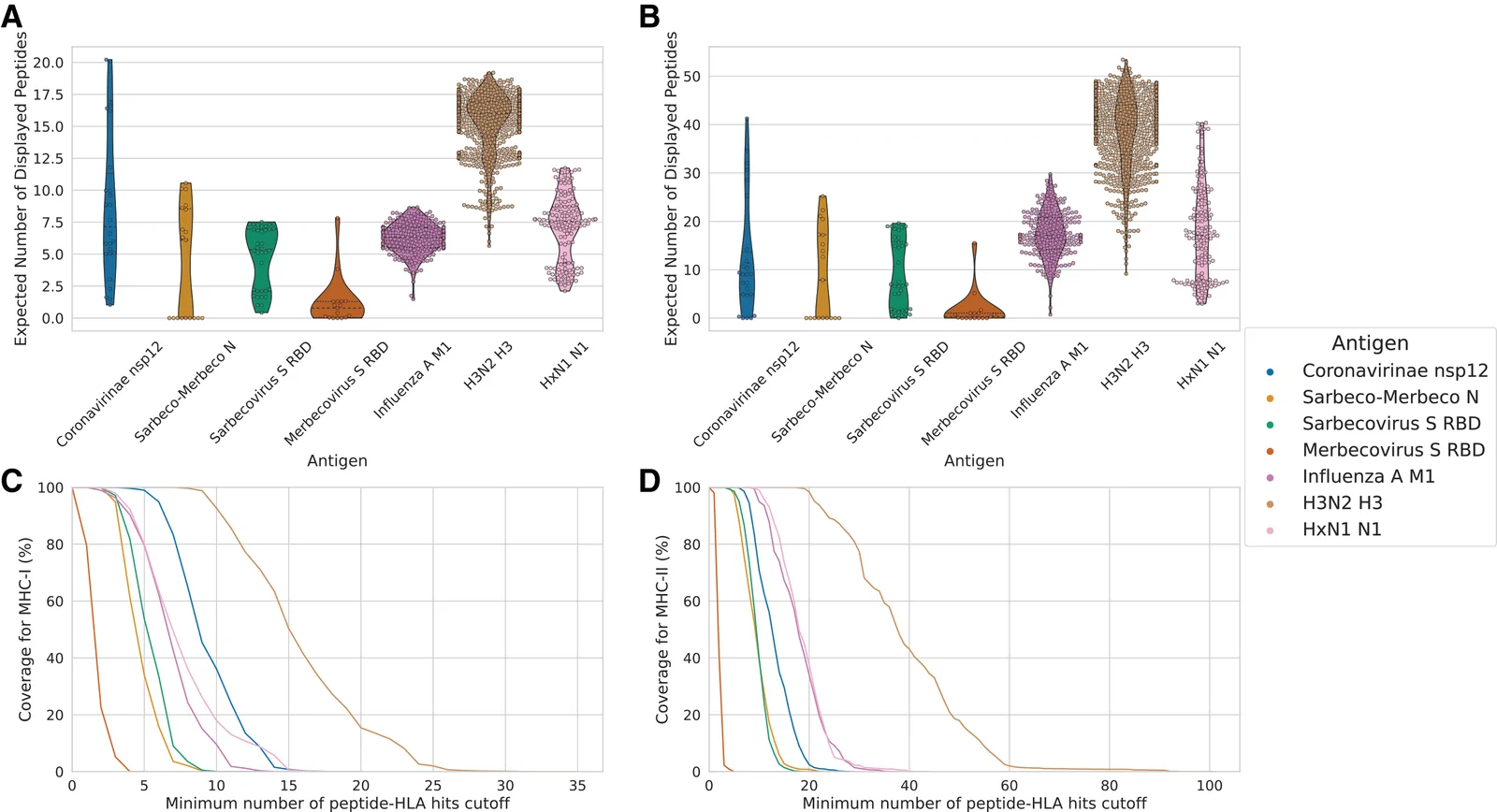
Generalizability of Spectravax to additional pathogens To demonstrate the generalisability of Spectravax, we applied the method to design six additional vaccine antigens from two diverse clades of viruses: Influenza A viruses and *Orthocoronavirinae* representing realistic targets for broad-spectrum vaccine design. All antigens were designed using default parameters (Supplementary Table S4). (A and B) Violin plots showing the E[#DPs] of the Spectravax antigen designs for (A) MHC-I and (B) MHC-II. The *x* axis shows the different antigens, and the *y* axis shows the expected number of displayed peptides. Each point represents a different pathogen in the target sequences. (C and D) Line plots showing the coverage of the average host population for (C) MHC-I and (D) MHC-II. The *x* axis shows the minimum number of peptide-HLA hits cutoff (*n*), and the *y* axis shows the coverage, which is the fraction of the host population predicted to display at least *n* peptides.

To demonstrate the generalizability of Spectravax beyond *Betacoronaviruses*, we additionally applied the method to design six vaccine antigens from two diverse clades of viruses, influenza A viruses and *Orthocoronavirinae*, spanning both internal and surface protein targets (Figure 6; Table S3). Spectravax produced antigens with high predicted host and pathogen coverage across all targets, supporting the method’s applicability to a broad range of broad-spectrum vaccine design problems.

**Figure 7:**
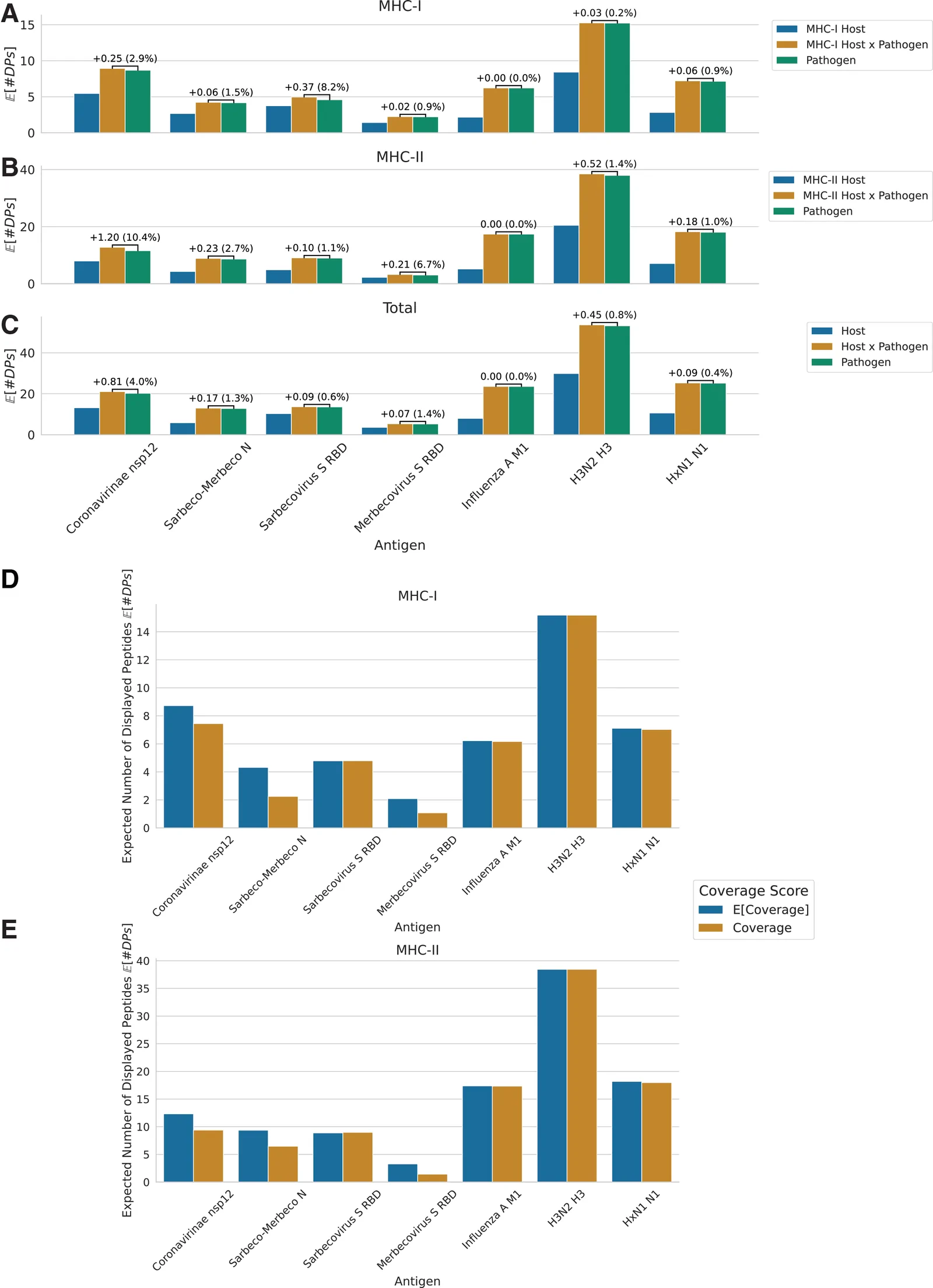
Decomposition of host and pathogen coverage in the expected number of displayed peptides (A–C) Attribution of host and pathogen coverage. For each of the seven *Coronavirinae* and influenza A antigens (*x* axis), three antigens were designed using Spectravax with different coverage score configurations: host coverage only (blue), pathogen coverage only (green), and the combined host × pathogen coverage (yellow). The expected number of displayed peptides (E[#DPs]) is shown on the *y* axis. Images show the antigens designed and evaluated for (A) MHC-I, (B) MHC-II, and (C) both MHC classes combined. The results indicate that the pathogen coverage score is the primary contributor to E[#DPs], as antigens designed using pathogen coverage alone exhibit higher values than those using host coverage alone. However, antigens designed with the combined host × pathogen coverage achieve the highest E[#DPs] across all antigens and MHC types. The difference between the combined and pathogen-only designs, annotated on the figure, highlights the added value of including host coverage in antigen design, as implemented in Spectravax, compared to methods like Epigraph that consider only pathogen coverage. (D and E) Comparison of antigens designed using the coverage and expected coverage criteria. For each of the seven *Coronavirinae* and influenza A antigens (*x* axis), two antigens were designed using Spectravax with the following coverage scores: E[Coverage] (blue) and coverage (yellow). The expected number of displayed peptides (E[#DPs]) is shown on the *y* axis. Images show evaluations for (D) MHC-I and (E) MHC-II.

We then evaluated how host and pathogen coverage each contribute to vaccine antigen quality across diverse target datasets. Antigens designed with the combined host × pathogen score consistently produced the highest expected number of displayed peptides across seven coronavirus and influenza A target sets, with pathogen coverage as the primary contributor (Figures 7A–7C). Building on this, we refined the coverage metric to use the eluted ligand score directly as a probability rather than as a binary threshold (expected coverage; see STAR Methods). This refinement increased the expected number of displayed peptides by 0.6 (10.2%) for MHC-I and 1.1 (7.8%) for MHC-II (Figures 7D and 7E) and is the recommended default for future Spectravax designs.

## Discussion

Broad-spectrum vaccines are crucial for combating antigenically diverse pathogens with pandemic potential. Computational methods for designing single-antigen broad-spectrum vaccines offer a generalizable approach that, in theory, can be applied to any pathogen. In this work, we introduced Spectravax, a computational framework for designing broad-spectrum vaccines that accounts for the genetic diversity present in both pathogen and host populations. The Spectravax framework has a strong theoretical foundation and is intuitive, as the scores correspond to real-world quantities such as population coverage and the number of displayed peptides. This makes the results interpretable and circumvents the need for arbitrary weights used in other methods.^39^ Furthermore, the underlying framework is generalizable, allowing it to be easily extended by other researchers. To demonstrate its utility, we applied Spectravax to design an N vaccine antigen to target *Sarbecoviruses* and *Merbecoviruses*.

One of the main advantages of Spectravax compared to existing methods is its use of the host coverage score, which maximizes T cell epitope presentation across a target population. A larger independent dataset of allele frequencies from AFND was used to validate the consistency of the host coverage estimates. Rarer HLAs, which tend to be less well studied and thus have less confident binding predictions, receive less weight in the host coverage score, thereby increasing the global coverage. Antigens designed using Spectravax, such as the N antigen, were predicted to display peptides across a wide range of HLA alleles, leading to effective host coverage. This was demonstrated for populations from three ancestries and in 95 countries. Incorporating the host coverage score consistently improved the expected number of displayed peptides across multiple antigens, although pathogen coverage was found to be more important (Figures 7A–7C).

The observed enrichment of MHC-II-restricted epitopes, particularly among HLA-DRB1 alleles, suggests that the predicted immune recognition may predominantly involve CD4^+^ helper T cell responses. Such responses are critical for orchestrating adaptive immunity, including cytokine production and B cell activation, which may enhance antibody-mediated protection.

Spectravax differs from existing methods in how it handles the relationship between host and pathogen coverage. The IEDB population coverage tool computes 1× host coverage, estimating whether an individual has at least one peptide-HLA hit, but does not account for the benefits of presenting multiple peptides, which can enhance immune responses and reduce the chance of viral escape. OptiVax-Robust^15^ uses conservation as a binary pre-filter, restricting candidates to fully conserved peptides before optimizing host coverage separately. This approach is effective for a single species such as SARS-CoV-2, where many peptides are fully conserved, but does not extend to cross-subgenus designs where few peptides pass a strict conservation threshold. Dorigatti and Schubert^37^ evaluated conservation and population coverage as separate metrics for their mosaic vaccine designs but did not combine them into a joint objective and did not provide experimental validation. Spectravax addresses these limitations by integrating pathogen coverage as a continuous score directly into the objective function, jointly optimizing it with host coverage in a single step.

Another major improvement over existing methods is the use of clade equalization, which helps mitigate biases toward clades with a higher number of characterized members, such as the *Sarbecovirus* subgenus in the case of *β*-CoVs. Interestingly, this process resulted in a chimera-like N antigen combining sequences from the *Merbecovirus* and *Sarbecovirus* clades at the N and C termini, respectively. This design was found to be representative of both subgenera and was the only evaluated antigen able to display peptides across all target sequences. This broad coverage was experimentally validated in mice using representatives of SARS-CoV, SARS-CoV-2, and MERS-CoV N proteins. Although the Epigraph and SARS-CoV-2 antigens induced stronger SARS-CoV and SARS-CoV-2 responses, the response against MERS-CoV was not statistically significant. Furthermore, we were able to identify the peptides responsible for the difference in response to MERS-CoV. Evaluation of the protein fitness landscape (Figure 4F) illustrates the trade-off between potency and breadth, an observation made with some of our earlier vaccine designs as well.^10^

As a DNA vaccine, the antigen is expressed by the host cell’s translational machinery, and complete protein folding is not required for T cell epitope presentation, as the antigen undergoes proteasomal degradation prior to MHC loading.

In terms of future work, Spectravax can be applied to other pathogens and host species, as evidenced by its application to seven different antigens from two diverse viral families (Table S3), using the improved coverage metric (Figures 7D and 7E). The pathogen coverage score could be improved by incorporating pathogen prevalence^13^ or spillover risk,^44^ similar to clade equalization. The host coverage score could incorporate predictions for T cell activation,^45^^,^^46^^,^^47^ in addition to peptide presentation. Although the N antigen demonstrated significant antibody responses, further computational optimization could be done to enhance these, either by integrating methods for linear B cell epitope prediction^18^ or by using generative deep learning models to design structurally conserved antigens while optimizing for both host and pathogen coverage.^17^ Additionally, *in vivo* studies could be conducted to evaluate the protective efficacy of the Spectravax N antigen against viral challenge.

The primary goal of Spectravax N is to elicit broad T cell immunity for the reduction of disease severity, rather than sterilizing immunity through neutralizing antibodies. Spectravax N could be combined with surface antigens such as S to elicit both neutralizing antibody and T cell responses; indeed, preclinical data from our group have demonstrated protection using combined S and N antigens against *Sarbecoviruses*,^48^ motivating the development of a broader N antigen as presented here.

In conclusion, Spectravax N is a T cell-focused computationally designed antigen that elicits immune responses against SARS-CoV, SARS-CoV-2, and MERS-CoV, highlighting its potential as a pan-*β*-CoV vaccine candidate. Apart from Spectravax N, we are aware of only one other single-antigen vaccine, consisting of a SARS-CoV-2 S protein with a MERS-CoV RBD, that has been shown to protect against SARS-CoV, SARS-CoV-2 Omicron variant, and MERS-CoV challenge in mice.^49^ The Spectravax method provides a substantial advancement in the rational design of broad-spectrum vaccines that can be applied to other pathogens and host species. The insights gained from this work regarding host and pathogen coverage offer valuable lessons for the broader field of vaccine development and can contribute to improved pandemic preparedness by supporting the development of vaccines that can protect against emerging infectious diseases.

### Limitations of the study

Limitations of the Spectravax method primarily stem from the underlying sequencing and immunological data used. The dbMHC frequency data used to estimate host coverage cover only 15 geographic regions and may not fully reflect the global human population.^50^ Mass spectrometry data are limited to the peptides that are eluted, which may not represent the full spectrum of peptides displayed *in vivo* and may be biased toward more common HLAs.^14^ Pathogen sequence databases are known to have biases such as over-representation of certain viral families, geographic regions, hosts, or time periods.^51^ The method also requires substantial computational resources for peptide-MHC binding predictions. Fortunately, the data limitations of Spectravax may diminish over time as more sequencing and immunological data become available. The experimental validation presented here measured T cell responses and antibody titers as proxies for vaccine-induced immunity; whether these responses confer protection against infection or disease would require confirmation through viral challenge studies.

## Resource availability

### Lead contact

Requests for further information, resources, and reagents should be directed to and will be fulfilled by the lead contact, Sneha Vishwanath (sv446@cam.ac.uk).

### Materials availability

This study did not generate new unique reagents.

### Data and code availability

• Reference data files required to run the pipeline, including HLA allele lists, haplotype frequencies, and the human reference proteome, are available on Zenodo (DOI: https://doi.org/10.5281/zenodo.18761002).
• Source code for Spectravax is available at https://github.com/PhilPalmer/Spectravax and archived on Zenodo (DOI: https://doi.org/10.5281/zenodo.18761421). The repository README provides command-line and Python API examples for running the core vaccine design pipeline.
• Any additional information required to reanalyze the data reported in this study is available from the lead contact upon request.

## Acknowledgments

P.P. was supported by a fellowship provided by Coefficient Giving (formerly Open Philanthropy). J.L.H. was supported by 10.13039/501100006041Innovate UK for COVID-19 vaccine program. We thank Dr. Leo C. James for the provision of purified recombinant SARS-CoV-2 nucleoprotein.

## Author contributions

P.P. and S.V. conceptualized the study, developed the Spectravax method, designed the antigens, and generated the figures. P.P., S.V., S.F., G.W.C., and J.L.H. planned the experiments. J.L.H. acquired funding. S.V., S.F., G.W.C., and J.L.H. supervised the project. C.G., S.P., and P.P. performed DNA purification and preparation. L.O. and G.W.C. performed the animal work. S.P., C.G., J.H., L.W., and S.F. performed the *in vitro* assays. S.F. and J.H. analyzed the assay data. P.P. wrote the original draft of the manuscript. S.V., G.W.C., S.F., and J.L.H. reviewed and edited the manuscript. All authors provided feedback on the paper.

## Declaration of interests

S.F., S.P., J.H., and J.L.H. are employees or shareholders of DIOSynVax Ltd.

## Declaration of generative AI and AI-assisted technologies in the writing process

During the preparation of this work, the authors used ChatGPT (OpenAI), GitHub Copilot (Microsoft/OpenAI), and Claude (Anthropic) in order to assist with manuscript writing and editing. After using these tools, the authors reviewed and edited the content as needed and take full responsibility for the content of the publication.

## STAR★Methods

### Key resources table

**Table:** 

REAGENT or RESOURCE	SOURCE	IDENTIFIER
**Antibodies**		

CD4 Monoclonal Antibody (RM4-5) APC	ThermoFisher Scientific	Cat#17-0042-83; RRID:AB_469323
CD3e Monoclonal Antibody (145-2C11) PE-Cyanine5.5	ThermoFisher Scientific	Cat#35-0031-82; RRID:AB_11219266
CD8a Monoclonal Antibody (53–6.7) PE-Cyanine7	ThermoFisher Scientific	Cat#25-0081-82; RRID:AB_469584
IFN-γ Monoclonal Antibody (XMG1.2) Alexa Fluor 488	ThermoFisher Scientific	Cat#53-7311-82; RRID:AB_469932
TNF-α Monoclonal Antibody (MP6-XT22) PE-eFluor 610	ThermoFisher Scientific	Cat#61-7321-82; RRID:AB_2574666
HRP-conjugated goat anti-mouse IgG (H + L) secondary antibody	Jackson ImmunoResearch	Cat#115-035-003; RRID:AB_10015289

**Biological samples**		

SARS-CoV recombinant full-length nucleocapsid protein	ACROBiosystems	Cat#NUN-S5229
SARS-CoV-2 recombinant full-length nucleocapsid protein	Kind gift from Dr. Leo C. James (MRC Laboratory of Molecular Biology, Cambridge)	N/A
MERS-CoV recombinant full-length nucleocapsid protein	CD Creative Diagnostics	Cat#DAG-H10295

**Chemicals, peptides, and recombinant proteins**		

SARS-CoV-2 N protein predicted epitope 15-mer peptide pool (11 aa overlap)	GenScript	N/A
SARS-CoV-2 N protein full-length 15-mer overlapping peptide pool (11 aa overlap)	GenScript	N/A
SARS-CoV N protein predicted epitope 15-mer peptide pool (11 aa overlap)	GenScript	N/A
SARS-CoV N protein full-length 15-mer overlapping peptide pool (11 aa overlap)	GenScript	N/A
MERS-CoV N protein predicted epitope 15-mer peptide pool (11 aa overlap)	GenScript	N/A
MERS-CoV N protein full-length 15-mer overlapping peptide pool (11 aa overlap)	GenScript	N/A
Histopaque 1083	Sigma-Aldrich	Cat#10831
Fixable Viability Dye eFluor 780	ThermoFisher Scientific	Cat#65-0865-14
Protein Transport Inhibitor (Brefeldin A + Monensin)	ThermoFisher Scientific	Cat#00-4980-03
eBioscience Cell Stimulation Cocktail (CSC)	ThermoFisher Scientific	N/A
UltraComp eBeads Compensation Beads	ThermoFisher Scientific	N/A
CytoFix/CytoPerm	BD Biosciences	Cat#554714
DMSO Hybri-Max	Sigma-Aldrich	N/A

**Critical commercial assays**		

Mouse IFN-γ Single-Color ELISpot kit	ImmunoSpot (CTL)	N/A
EndoFree Plasmid Mega Kit	Qiagen	N/A
Pierce LAL Chromogenic Endotoxin Quantitation Kit	Thermo Scientific	N/A

**Deposited data**		

Spectravax reference data files (HLA allele lists, haplotype frequencies, human reference proteome)	This paper; Zenodo	DOI: https://doi.org/10.5281/zenodo.18761002
Spectravax source code	This paper; Zenodo	DOI: https://doi.org/10.5281/zenodo.18761421;https://github.com/PhilPalmer/Spectravax
Sarbecovirus and Merbecovirus nucleocapsid protein sequences used for vaccine design	Benson et al.^36^; NCBI Virus	taxid:2509511 (Sarbecovirus); taxid:2509494 (Merbecovirus)

**Experimental models: Cell lines**		

Mouse: C57BL/6J: C57BL/6J	Charles River Laboratories (Margate, UK)	RRID:IMSR_JAX:000664

**Recombinant DNA**		

Spectravax N antigen codon-optimised gene in pEVAC vector	This paper; GeneArt, Thermo Fisher Scientific	N/A
Epigraph N antigen codon-optimised gene in pEVAC vector	This paper; GeneArt, Thermo Fisher Scientific	N/A
SARS-CoV-2 N wild-type codon-optimised gene in pEVAC vector	This paper; GeneArt, Thermo Fisher Scientific	N/A
MERS-CoV N wild-type codon-optimised gene in pEVAC vector	This paper; GeneArt, Thermo Fisher Scientific	N/A

**Software and algorithms**		

Spectravax	This paper	DOI: https://doi.org/10.5281/zenodo.18761421;https://github.com/PhilPalmer/Spectravax
NetMHCpan v4.1/NetMHCIIpan v4.0	Reynisson et al.^14^; Nilsson et al.^52^	https://services.healthtech.dtu.dk/services/NetMHCpan-4.1/
EvalVax-Robust (OptiVax)	Liu et al.^15^	N/A
Epigraph	Theiler et al.^13^	N/A
MAFFT v7	Katoh et al.^38^	https://mafft.cbrc.jp/alignment/software/
CD-HIT	Li et al.^37^	https://github.com/weizhongli/cdhit
scikit-learn	Pedregosa et al.^53^	https://scikit-learn.org/
SciPy	Virtanen et al.^54^	https://scipy.org/
Matplotlib	Hunter, 2007^55^	https://matplotlib.org/
FlowJo v10.9.0	BD Biosciences	https://www.flowjo.com/
redun	Rasmussen et al.^56^	https://github.com/insitro/redun
GeneOptimizer	Raab et al.^57^; Thermo Fisher Scientific	N/A

**Other**		

MACS SmartStrainers (70 μm)	Miltenyi Biotec	N/A
NanoDrop spectrophotometer	Thermo Scientific	N/A
ELISpot reader S6 Ultra M2	ImmunoSpot (CTL)	N/A
Attune NxT Flow Cytometer (3 laser)	ThermoFisher Scientific	N/A
BD LSRFortessa Cell Analyser (5 laser)	BD Biosciences	N/A
BioTek 800TS microplate reader	BioTek	N/A
Nunc MaxiSorp flat-bottom 96-well plates	ThermoFisher Scientific	N/A

### Experimental model and study participant details

#### Mouse immunisation

Four groups of twelve 7–10-week-old female C57BL/6J mice, plus an additional six mice for the control group, were obtained from Charles River Laboratories (Margate, U.K). All animal experiments were conducted in compliance with institutional guidelines and approved by the Animal Welfare and Ethical Review Board covering University Biomedical Services, University of Cambridge. Mice were immunised subcutaneously in the rear flank with a 40 *μ*g dose of plasmid DNA diluted in phosphate-buffered saline (PBS). Immunisations were administered on days 0, 28, and 56, following the schedule shown in Figure 5A. Terminal blood collection was performed by cardiac puncture under non-recovery isofluorane anesthesia on day 84, and spleens were harvested using aseptic technique for immunological assays.

### Method details

#### Spectravax framework

Spectravax is a computational method to design broad-spectrum vaccines by maximising the coverage of both pathogen and host populations, accounting for genetic diversity across both.

The vaccine design problem is formulated as follows^13^^,^^37^:

Let *S* = {*s*_1_,*s*_2_, …,*s*_*N*_} represent the set of protein sequences from the target pathogen population. Each protein sequence *s*_*i*_ is split into overlapping k-mers of different lengths from a set *K* = {*k*_1_,*k*_2_, …,*k*_|*K*|_}. The union of all k-mers in the pathogen sequences is denoted as *M*(*S*). The objective is to design a contiguous protein sequence *v* composed of k-mers from *M*(*S*) that maximises the total coverage score, subject to the constraint shown below. The constraint ensures that any two consecutive *k*-mers in the vaccine design share an overlap of *k*-1 amino acids, where *k* is the shorter *k*-mer length. This ensures contiguity of the amino acid sequence while accommodating varying *k*-mer lengths.

The optimisation problem is expressed as

(Equation 1) $$\text{maximise}\;\sum_{m\in M_{v}}cov\left(m\right)$$

subject to overlap(*m*_*i*-1_,*m*_*i*_) = *min*(*k*(*m*_*i*-1_),*k*(*m*_*i*_))-1,∀*i*∈{2, …,|*M*_*v*_|}.where $\text{overlap}\left(m_{i-1},m_{i}\right)=\max\left\{l:m_{i-1}\left[\left|m_{i-1}\right|-l:\right]=m_{i}\left[:l\right]\right\}\text{.}$

Where *M*_*v*_ is the set of k-mers in the vaccine design *v*, and *cov*(*m*) is the total coverage score for k-mer *m*, which is calculated as the product of the pathogen and host coverage scores:

(Equation 2) $$cov\left(m\right)=cov_{\text{host}}\left(m\right)\times cov_{\text{path}}\left(m\right)\text{.}$$

#### Pathogen population coverage

The pathogen coverage score (*cov*_path_) is calculated as the fraction of target pathogen sequences containing the k-mer^13^:

(Equation 3) $$cov_{\text{path}}\left(m\right)=\frac{n_{m}}{N}\text{,}$$

where *n*_*m*_ is the number of target sequences that contain the k-mer *m*, and *N* is the total number of target sequences. This gives a score between 0 and 1, where 1 indicates that the k-mer is fully conserved across the target pathogen population. This is also referred to as conservation in the wider literature,^37^ however, we use the term pathogen coverage to emphasise similarities with the host coverage score.

#### Host population coverage

The host coverage score (*cov*_host_) is computed as the weighted sum of the MHC-I (*cov*_mhc1_) and MHC-II (*cov*_mhc2_) coverage scores:

(Equation 4) $$cov_{\text{host}}\left(m\right)=w_{\text{mhc}1}\times cov_{\text{mhc}1}\left(m\right)+w_{\text{mhc}2}\times cov_{\text{mhc}2}\left(m\right)\text{,}$$

where *w*_mhc1_ and *w*_mhc2_ are the respective weights for MHC-I and MHC-II scores, set to 1 unless specified otherwise. This term is often simply referred to as population coverage in the literature,^15^ however, we use the term host coverage to differentiate it from the pathogen coverage score.

The MHC-I and MHC-II coverage scores are calculated using the EvalVax-Robust algorithm,^15^ which accounts for linkage disequilibrium between HLA alleles by using haplotype frequencies. The method combines peptide-MHC binding predictions with host HLA allele frequencies to estimate the proportion of the host population that can present each k-mer. Host HLA allele frequencies were collected from the dbMHC database, covering three main ancestries (Asian, African, and European).^50^ For validation across broader populations, allele frequencies from the Allele Frequency Net Database (AFND) covering 95 countries were also used.^41^^,^^56^ However, unlike EvalVax-Robust,^15^ we use the eluted ligand (EL) binding score as the binding criteria, as described below. This enabled the use of the expected host coverage score, which increased the expected number of displayed peptides (see *Expected host coverage score* below).

#### Expected host coverage score

As the EL score is essentially a measure of the likelihood that a peptide is naturally processed and presented on MHC molecules, it can be incorporated directly as a probability in the calculation of host coverage. Unlike the binary hit-based approach used by EvalVax-Robust,^15^ where peptides are filtered based on a strict binding affinity threshold, this probabilistic approach uses the EL scores to derive *P*_*p*,*a*_, the probability that allele *a* presents peptide *p*. For each allele *a*, let:

$$P_{p,a}=\text{EL}\left(p,a\right)\text{.}$$

#### Given a haplotype *h* = *A*_*i*_*B*_*j*_*C*_*k*_, the probability that peptide *p* is displayed is

$$P_{p,h}=1-\prod_{a\in \left\{A_{i},B_{j},C_{k}\right\}}\left(1-P_{p,a}\right)\text{.}$$

Rather than applying a threshold to determine whether a haplotype is covered by at least one peptide, the probabilities *P*_*p*,*h*_ are summed over all peptides in *O*:

$$\sum_{p\in O}P_{p,h}\text{.}$$

This summation provides the expected number of displayed peptides per haplotype, reflecting the average count of peptides likely to be presented rather than the probability that at least one peptide is presented. When weighting by haplotype frequencies and summing over all haplotypes, we obtain:

$$\sum_{i=1}^{\left|A\right|}\sum_{j=1}^{\left|B\right|}\sum_{k=1}^{\left|C\right|}G\left(i,j,k\right)\sum_{p\in O}P_{p,h}\text{.}$$

This value represents the expected number of displayed peptides per individual in the population.

If we focus on a single peptide *p*, then:

$$\sum_{i=1}^{\left|A\right|}\sum_{j=1}^{\left|B\right|}\sum_{k=1}^{\left|C\right|}G\left(i,j,k\right)P_{p,h}$$

directly yields the expected fraction of the population that displays that peptide.

This indicates that expected host coverage provides a more sensitive measure for evaluating individual k-mers. Under the strict thresholding from OptiVax, many peptides have zero 1-times host coverage. In contrast, the expected host coverage is typically non-zero, enabling improved discrimination among candidate peptides.

#### Peptide-MHC binding predictions

Binding predictions were performed using the deep learning methods NetMHCpan v4.1 for MHC-I and NetMHCIIpan v4.0 for MHC-II.^14^ The eluted ligand (EL) score was selected as the binding criteria, as it was shown to be discriminative in identifying binders for SARS-CoV-2 peptides and well calibrated (Figure S6).^15^ Peptide-HLA binding predictions were made for all relevant HLA alleles from the dbMHC database.^50^ Due to the computational intensity of these predictions, calculations were parallelised over both k-mers and HLA alleles using the redun workflow management system.^53^

#### Sequence data and processing

Protein sequences of the nucleocapsid (N) protein from the *Sarbecovirus* (taxid: 2509511) and *Merbecovirus* (taxid: 2509494) subgenera were retrieved from the NCBI Virus database.^30^ Sequences were filtered to include only entries with the relevant protein names: “nucleocapsid phosphoprotein”, “nucleocapsid protein”, “nucleoprotein”, and “nucleocapsid”. Sequences with incomplete or ambiguous amino acids were removed. To reduce redundancy and ensure a representative set of sequences, nucleotide sequences were clustered using CD-HIT^31^ at a threshold of 99% identity, translated to amino acids and duplicate protein sequences were removed. After preprocessing, 84 target sequences remained for vaccine design. This reflects the limited number of unique, high-quality nucleocapsid sequences available for non-SARS-CoV-2 *Betacoronaviruses* in public databases. Pairwise amino acid identity ranged from 83 to 100% within *Sarbecoviruses* and 71–100% within *Merbecoviruses* (Figure S3).

#### Antigen design

We applied Spectravax to design a vaccine antigen targeting the *Betacoronavirus* (*β*-CoV) nucleocapsid (N) protein. The sequences were first clustered at 95% identity within Spectravax, resulting in a final set of 19 target sequences. To visualise the sequence diversity across the N protein, we generated a Weblogo for the entire length of N protein for the 19 sequences in our dataset (Figure S7).

Clade equalisation was then employed to ensure balanced representation of the *Sarbecovirus* and *Merbecovirus* subgenera. By default, Spectravax determines the number of clusters automatically by identifying the elbow point in a within-cluster sum of squares (WCSS) plot, which measures the compactness of clusters as a function of their number. For the N antigen, we swept cluster values from 1 to 19 and fixed the number at seven, as this produced a balanced design incorporating conserved elements from both subgenera (Figures 3B and 4E).

The MHC-I weight was set to double that of MHC-II, in an attempt to focus on eliciting a CD8^+^ T cell response. All other parameters were set to their default values. For all other vaccine designs (Figure 6), the default parameters were used (Table S4).

#### Computational analyses

The expected number of displayed peptides (E[#DPs]) is computed by summing over all thresholds of *n* displayed peptides, weighted by the corresponding coverage at each threshold^15^:

(Equation 5) $$E\left[\#\text{DPs}\right]=\sum_{n=1}^{N_{\max}}\left(n\times cov\right)\text{,}$$

where *cov* is the coverage, i.e., the fraction of the population that is predicted to display exactly *n* peptides, and *N*_*max*_ is the maximum possible number of peptides.

Principal component analysis (PCA) was performed to visualise the diversity of pathogen sequences and to position the vaccine designs within sequence space. A multiple sequence alignment (MSA) was first generated using MAFFT.^32^ The MSA is then converted to a numerical matrix where the amino acids at each position are ranked by their frequency in the MSA. PCA was applied to this feature matrix to reduce dimensionality, using the Python package scikit-learn^57^ and the first two principal components were plotted.

#### Plasmid DNA preparation

Genes encoding the antigens were commercially synthesised and cloned into the pEVAC vector (GeneArt, Thermo Fisher Scientific). The N sequences were codon-optimised for expression in mammalian cells using the GeneOptimizer algorithm,^52^ which includes screening for cryptic splice sites, extreme GC content, and other sequence elements that could compromise mRNA stability and expression fidelity. Post-translational modifications such as N- and O-linked glycosylation are not expected to affect the expressed antigen, as the nucleocapsid protein localises to the cytoplasm and does not enter the secretory pathway where glycosylation occurs. Plasmid DNA used for vaccination were outgrown and isolated using EndoFree Plasmid Mega Kit (Qiagen) following the manufacturer’s instructions. The extracted plasmids were then quantified using UV spectrophotometry with a NanoDrop device (Thermo Scientific).^52^ The presence of endotoxin was measured using the Pierce LAL Chromogenic Endotoxin Quantitation Kit (Thermo Scientific) and was found to be below the detection limit of 20 EU/mL. The sequences were confirmed using Sanger sequencing (Source BioScience).

#### Peptide design and synthesis

Peptide sequence predictions were generated using NetMHCpan and NetMHCIIpan software without a length restriction setting.^14^^,^^54^ For predicted peptide pools, the library of peptides was filtered for binding values, number of alleles being targeted, and the number of times amino acid was predicted to be a part of the peptide. Once such assembly of peptides was selected, the predicted peptides were aligned to the reference sequence and a peptide library of 15-mer with overlap of 11-mer was generated.

For overlapping peptide pools, entire length of the protein reference sequence was divided into 15-mer peptides with 11-mer overlap. All peptides were synthesized by GenScript and reconstituted in 4% DMSO in PBS.

#### Isolation of murine splenocytes

Murine splenocytes were isolated from dissected spleens by mashing spleens on MACS SmartStrainers (Miltenyi Biotec) and layering them on the Histopaque 1083 reagent (SigmaAldrich). Puffy coat containing immune cells was collected from the phase separation region and washed in R10 medium (RPMI medium (Gibco) supplemented with 10% Fetal bovine serum (FBS) (Gibco)). Cell pellets were resuspended and frozen in FBS supplemented with 10% DMSO Hybri-Max (SigmaAldrich).

#### ELISpot assay

ELISpot assays with frozen splenocytes were performed according to the manufacturer’s protocol using the mouse IFN-*γ* Single-Color ELISpot kit (Immunospot). Frozen splenocytes were thawed, washed once with R10 medium and resuspended in CTL medium supplemented with L-glutamine at the desired cell density (in the range of 2-5x106 cells/mL). Custom made predicted peptides for full-length SARS-CoV-2 N (NCBI Reference Sequence: YP_009724397.2), SARS-CoV N (NCBI Reference Sequence: YP_009825051.1) and MERS-CoV N (NCBI Reference Sequence: YP_009047204.1) proteins were used. For the stimulation, 100 *μ*L of peptide solution in CTL at 4 *μ*g/peptide/mL were mixed with 100 *μ*L of cell suspension in the 96-well ELISpot plate and incubated for 24h in 37°C in humidified incubator with 5% CO2. As a positive control, eBioscience Cell stimulation cocktail (ThermoFisher Scientific) was used at 1× dilution. CTL medium was used as a non-stimulation control. The plates were developed as per manufacturer’s protocol, dried, scanned and analyzed on ELISpot reader S6 Ultra M2 (ImmunoSpot).

To identify immunodominant peptides for the MERS-CoV N protein, 8 smaller peptide pools were prepared and two samples from each Spectravax and Epigraph vaccinated groups of mice were assayed. After identification of the two specific immunogenic peptides (MN14 and MN15 from the original MERS-CoV N peptide pool), there were used at 2 *μ*g/mL for the final concentration on the entire available cohort of responding animals from Spectravax and Epigraph groups. All samples were assayed in at least technical duplicates.

#### Flow cytometry cytokine assay

Under sterile conditions murine splenocytes were placed into RPMI (10% FBS, 1% P/S 50 *μ*M beta-mercaptoethanol) media in a 96 well U-bottomed plate at a concentration of 1.5 × 106/mL, 200 *μ*L per well. The cells were incubated either with Protein Transport Inhibitor (Thermofisher 00-4980-03) + 1 *μ*g/mL SARS-CoV, SARS-CoV-2 or MERS-CoV pooled peptides. PTI and CSC were used as a negative and positive control respectively. Cells were incubated at 37°C for 6 h.

Cells were washed with FACS buffer (PBS, 0.5% BSA, 0.01% NaN3) three times, blocked with FACS buffer with 2% rat serum for 15 min at 4°C, then stained with Fixable Viability Dye eFluor 780 (ThermoFisher 65-0865-14), CD4 Monoclonal Antibody (RM4-5) APC (ThermoFisher 17-0042-83), CD3e Monoclonal Antibody (145-2C11) PE-Cyanine5.5 (ThermoFisher 35-0031-82) and CD8a Monoclonal Antibody (53–6.7) PE-Cyanine7 (ThermoFisher 25-0081-82). Cells were incubated for 30 min at 4°C in the dark, washed three times with FACS buffer, then resuspended in CytoFix/CytoPerm (BD Biosciences 554714) and left at room temperature (RT) in the dark for 15 min. Cells were then washed with perm/wash buffer 3 times, stained for intracellular cytokines with IFN-*γ* Monoclonal Antibody (XMG1.2) Alexa Fluor 488 (ThermoFisher 53-7311-82) and TNF-*α* Monoclonal Antibody (MP6-XT22), PE-eFluor 610 (ThermoFisher 61-7321-82) at RT in the dark for 30 min. Washed with perm/wash buffer 3 times, and finally resuspended in FACS buffer for analysis on an Attune NxT Flow Cytometer equipped with 3 lasers (ThermoFisher Scientific). A BD LSRFortessaTM Cell Analyser with 5 lasers (BD Biosciences) was used when analysing the individual reactions to MERS-CoV pools 1–8 at 2 *μ*g/mL each, and MERS peptides MN14 and MN15 at 2 *μ*g/mL each in a separate experiment. UltraComp eBeads Compensation Beads were used for single color compensation controls. Analysis was performed using FlowJo 10.9.0 (BD Biosciences).

#### ELISA assay

Serum IgG antibody binding responses were assessed by ELISA against recombinant full length N proteins from SARS-CoV (ACROBiosystems, NUN-S5229), SARS-CoV-2 (kind gift from Dr. Leo C. James), and MERS-CoV (CD Creative Diagnostics, DAG-H10295). Nunc MaxiSorp flat-bottom plates were coated with 1 *μ*g/mL of recombinant N protein in PBS (−/−) and incubated overnight at 4°C. Plates were blocked with 3% milk in PBST (PBS with 0.1% Tween 20) for 1 h at 300 RPM. 50 *μ*L of serum samples diluted in PBST with non-fat milk was added and incubated for 2 h at room temperature. Plates were washed three times with PBST, and HRP-conjugated goat anti-mouse IgG (H + L) secondary antibody (Jackson Immunoresearch, 115-035-003; 1:3000 dilution) was added to each well. After incubation for 1 h at room temperature, plates were washed three times with PBST, and 50 *μ*L of TMB substrate was added to each well. The reaction was stopped after three minutes by adding 50 *μ*L of 1 M H _2_ SO _4_, and the absorbance was read at 450 nm using a BioTek 800TS microplate reader.

### Quantification and statistical analysis

Statistical significance was determined using a two-sided Mann-Whitney *U* test for comparisons between groups using the SciPy library in Python.^55^ All plots were generated using the Matplotlib library in Python.^58^

## Supplemental information

Document S1. Figures S1–S7 and Tables S1–S4

Document S2. Article plus supplemental information
