# Supplementary material for: A computational method to design broad-spectrum T cell-inducing vaccines applied to Betacoronaviruses
Source: Cell Rep Methods. 2026 Jun 29;6(8):101508. doi: 10.1016/j.crmeth.2026.101508 (PMC13494552; doi:10.1016/j.crmeth.2026.101508)
Supplement: Supplementary file 1 [file mmc1.pdf]

Cell Reports Methods, Volume *6*

Supplemental information

A computational method to design broad-spectrum T cell-inducing vaccines applied to *Betacoronaviruses*

Phil Palmer, Sofiya Fedosyuk, Srivatsan Parthasarathy, Jonathan Holbrook, Charlotte George, Laura O'Reilly, Lara Wiegand, George William Carnell, Jonathan Luke Heeney, and Sneha Vishwanath

Supplementary Data

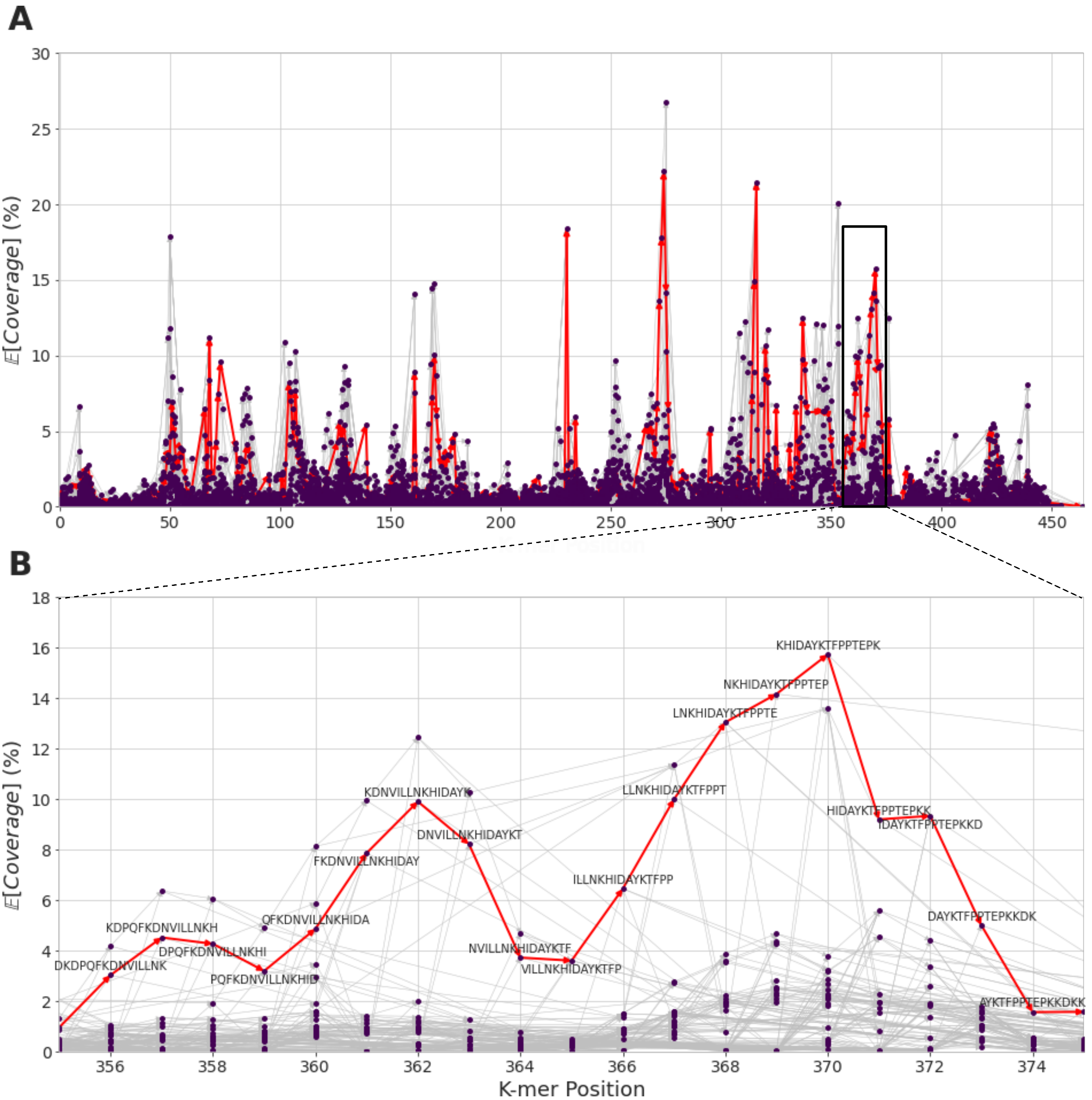

Figure S1. K-mer graph for the Spectravax N antigen design, Related to STAR Methods. (**A**) K-mer graph across the entire sequence. The section highlighted in black rectangle corresponds to the region shown in subplot B. (**B**) K-mer graph for amino acids 355-375. The nodes of the graph are the dots that represent all unique k-mers. The edges are the thin lines that connect k-mers whose sequence overlap by $k-1$ amino acids. The red lines represent the optimal path through the graph, which corresponds to the vaccine design. The x-axis represents the position in the sequence, the y-axis represents the expected coverage score $\mathbb{E}[cov]$ (STAR Methods). The position (x-axis) is approximate as a given k-mer can appear at different positions in different sequences. Therefore, the k-mer position was measured by the first instance found in a multiple sequence alignment of the target sequences generated using MAFFT[1].

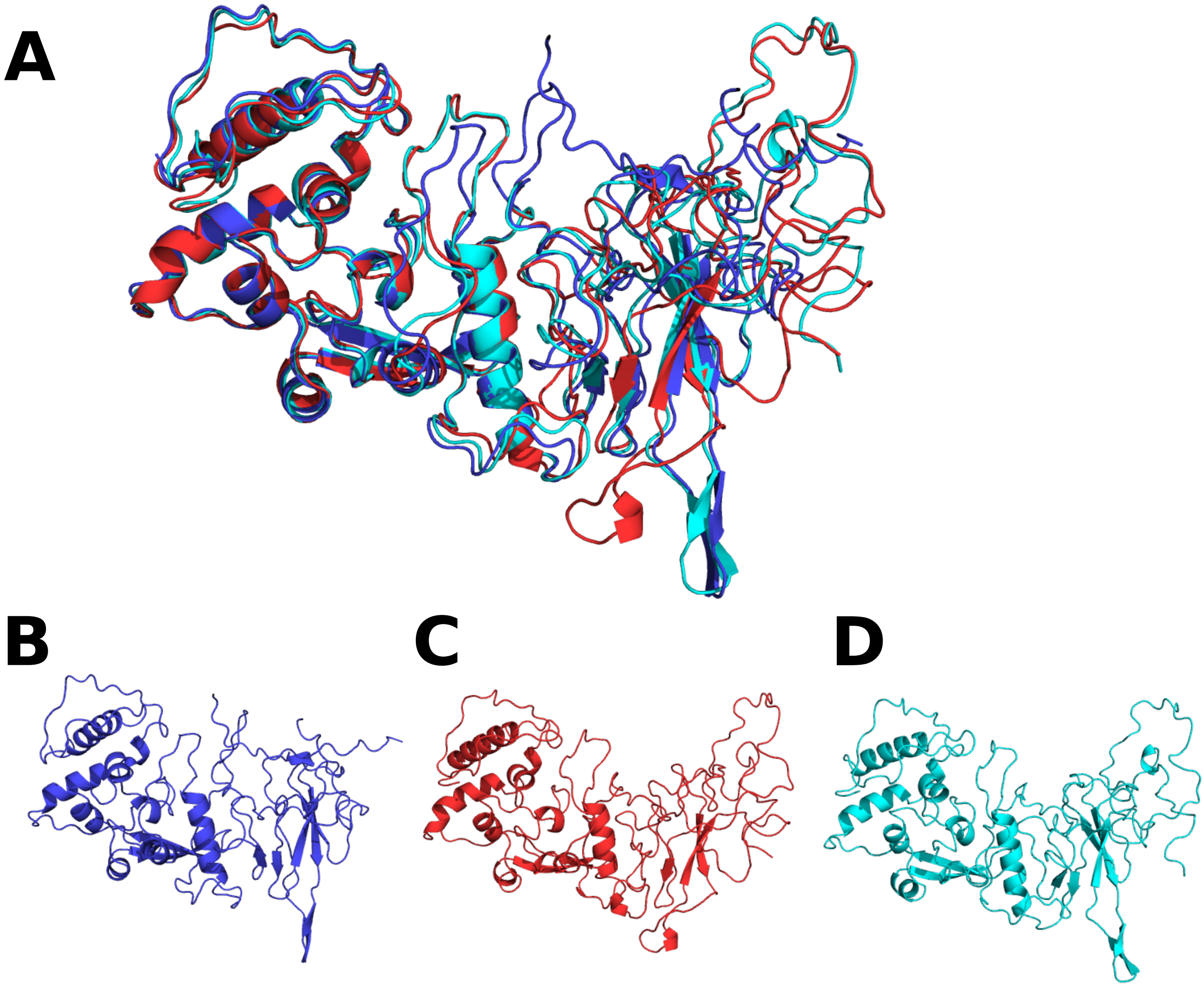

Figure S2. AlphaFold-predicted structures of Spectravax, Epigraph, and SARS-CoV-2 Nucleoproteins, Related to Figure 3. (**A**) Overlay of all structural models: (**B**) Spectravax N (blue), (**C**) SARS-CoV-2 N (red, PDB ID: 8FD5[2]), (**D**) Epigraph N (cyan). Spectravax and Epigraph N structures were predicted with ColabFold[3] using AlphaFold2[4] and the SARS-CoV-2 N template: 8FD5[2]. Default parameters were used and the best model was selected based on pTM-score.

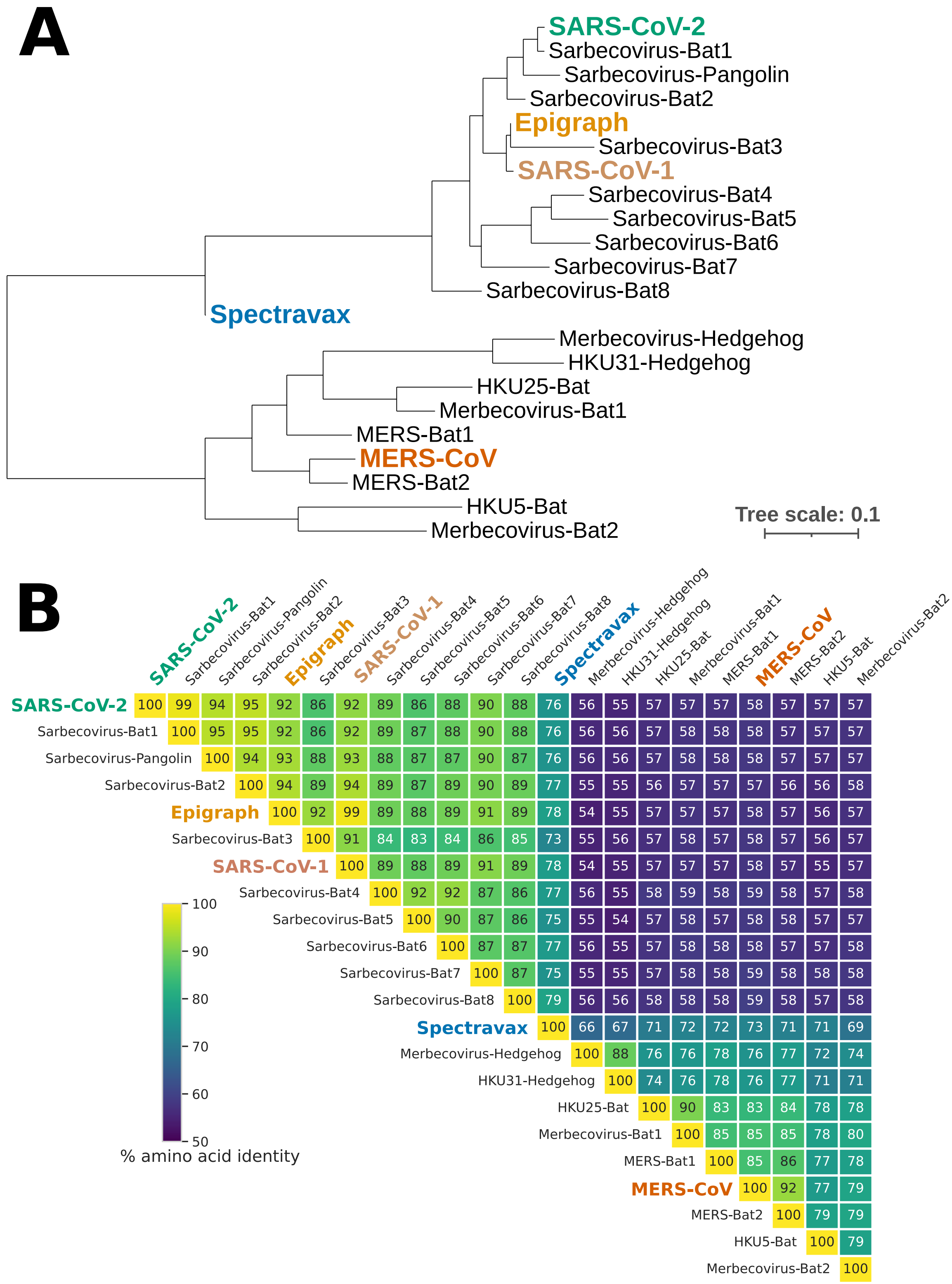

Figure S3. Phylogenetic tree and sequence identity matrix of *Sarbecovirus* and *Merbecovirus* Nucleoprotein sequences, Related to STAR Methods. (**A**) Phylogenetic tree produced by multiple sequence alignment (MAFFT[1]) and constructed in IQ-TREE 2[5] using the LG model[6], then visualised with iTOL[7] with midpoint root. (**B**) Sequence identity matrix generated by pairwise alignment using Biopython's `globalxx` method[8]. The Nucleoprotein sequences are those used as the target of vaccine design and supplemented with: Spectravax design (blue), Epigraph design (yellow), SARS-CoV-1 (brown), SARS-CoV-2 (green), and MERS-CoV (orange). The GenBank protein IDs for the target proteins are as follows: QSQ01658.1 (Sarbecovirus-Bat1), QVT76614.1 (Sarbecovirus-Pangolin), QZX47293.1 (Sarbecovirus-Bat2), QZX47283.1 (Sarbecovirus-Bat3), YP_003858591.1 (Sarbecovirus-Bat4), QVN46576.1 (Sarbecovirus-Bat5), QTJ30142.1 (Sarbecovirus-Bat6), BCG66635.1 (Sarbecovirus-Bat7), UFP05031.1 (Sarbecovirus-Bat8), YP_009513018.1 (Merbecovirus-Hedgehog), UMO75626.1 (HKU31-Hedgehog), ASL68949.1 (HKU25-Bat), AHY61344.1 (Merbecovirus-Bat1), AUM60021.1 (MERS-Bat1), ATQ39393.1 (MERS-Bat2), YP_001039969.1 (HKU5-Bat), ABG47058.1 (Merbecovirus-Bat2).

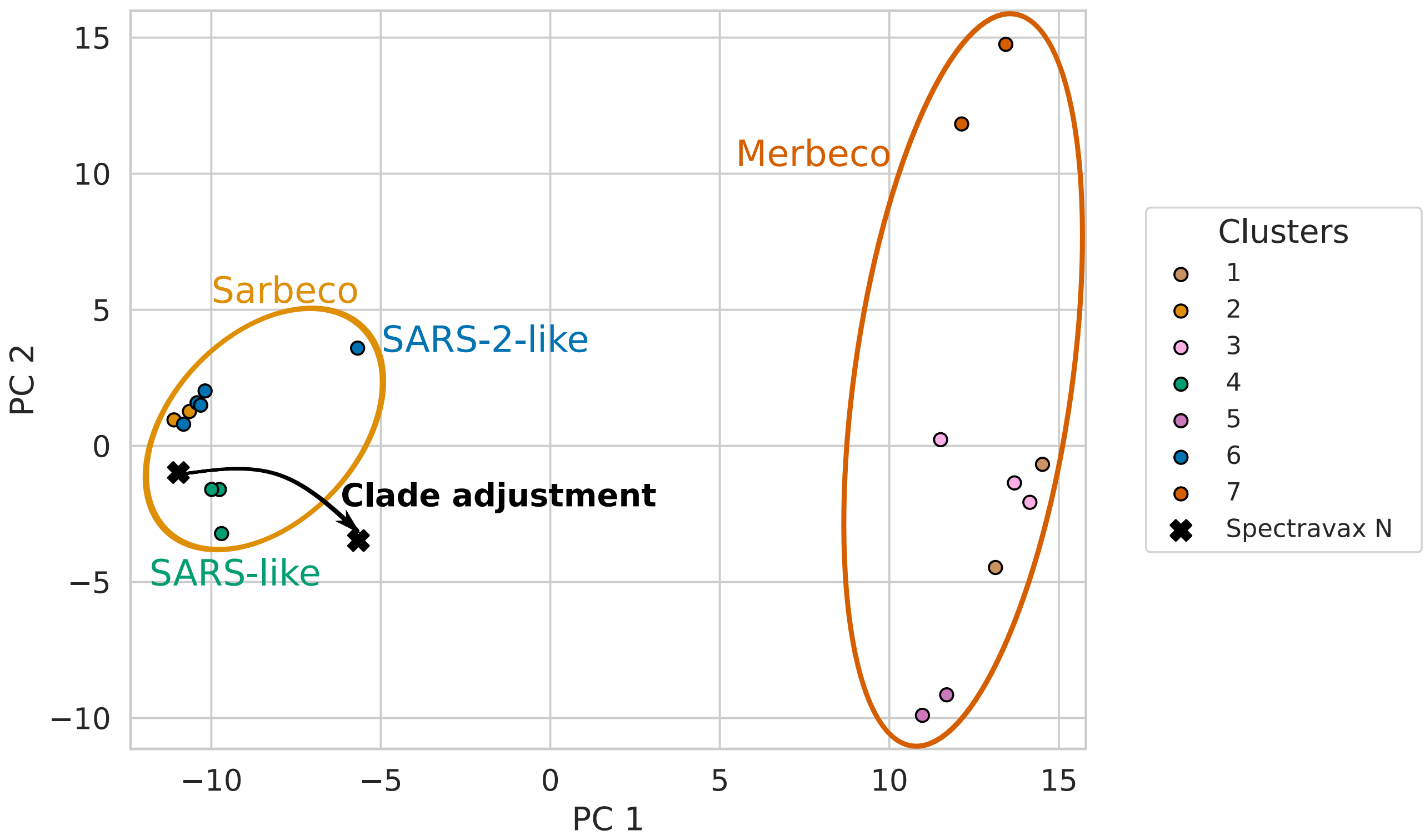

Figure S4. Spectravax clade adjustment, Related to STAR Methods. The effect of clade adjustment on the Spectravax N design is shown using principal component analysis (PCA). The x- and y-axes represent the first two principal components of the ranked multiple sequence alignment (MSA) of the *Sarbecovirus* and *Merbecovirus* N sequences. The clade adjustment process (shown as a black arrow) shifts the vaccine design closer towards the centre of the sequence space, enhancing its breadth across the target pathogen population. Clade adjustment therefore addresses bias towards overrepresented clades (e.g., *Sarbecovirus*) by adjusting coverage scores to give higher weight to k-mers from underrepresented clades (e.g., *Merbecovirus*).

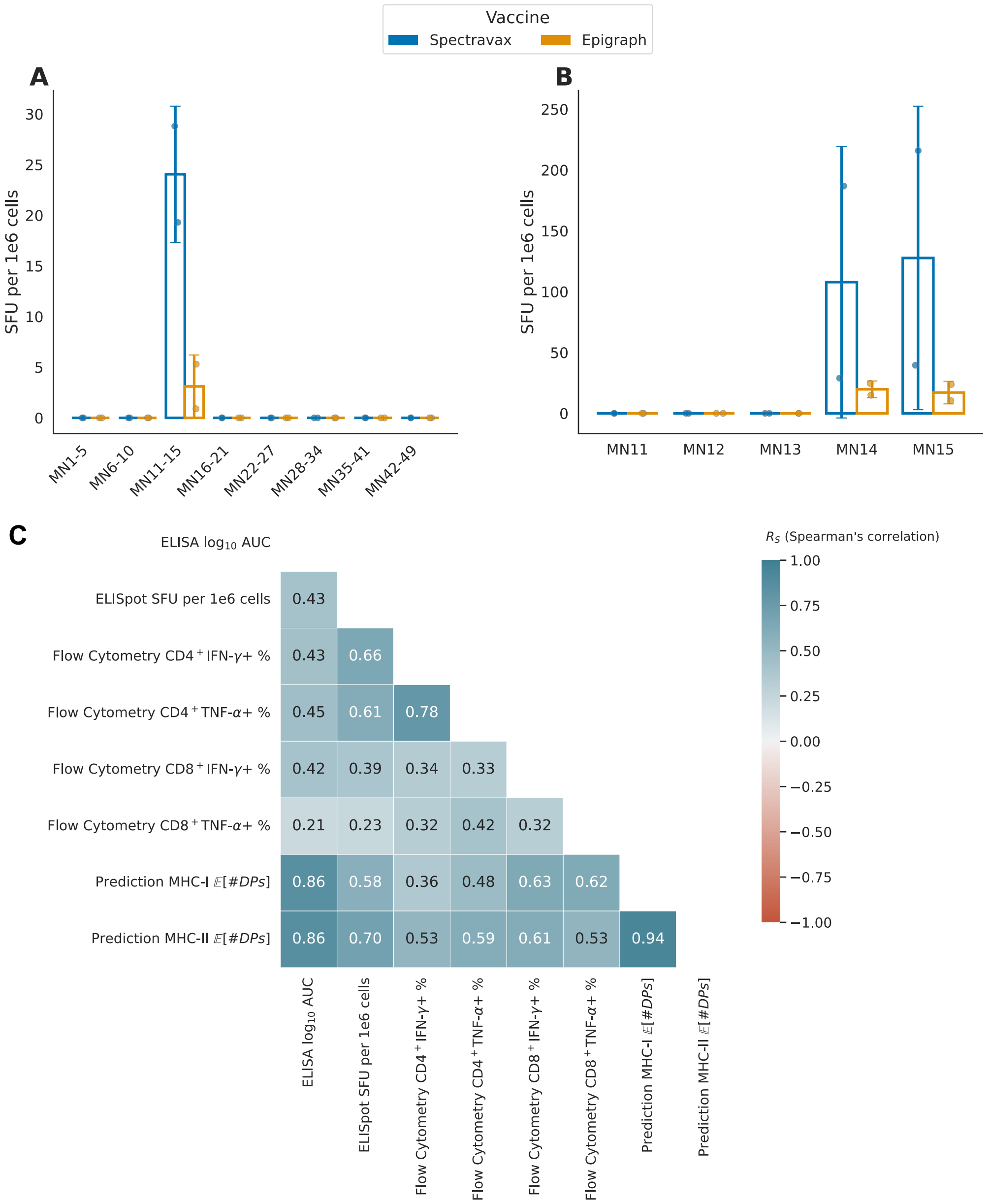

Figure S5. Identification of immunodominant peptides and correlation of experimental results with computational predictions, Related to Figure 5. (**A**) ELISpot stimulation with MERS-CoV Nucleocapsid (MN) peptide pools. (**B**) ELISpot stimulation with individual peptides from pool MN11–15 that showed the highest response. Two antigens were evaluated in (A) and (B): Spectravax (blue) and Epigraph (yellow, $n = 2$). (**C**) Correlation matrix of experimental results and computational predictions in C57BL/6J mice. The Spearman correlation coefficient (R_S) is shown for each pair of methods. The colour of each cell indicates the strength of the correlation, with blue representing positive correlations and red representing negative correlations. Only positive correlations were observed between the different assays, indicating general agreement in the strength of immune responses measured by different methods. ELISpot responses correlated more strongly with CD4$^+$ T-cell responses ($R_S > 0.6$) than with CD8$^+$ T-cell responses ($R_S < 0.4$), suggesting that CD4$^+$ cells were the main drivers of the T-cell responses. The experimental results also showed a strong correlation with the computational predictions. In particular, the computational predictions correlated strongly with the ELISA responses (MHC-I $R_S = 0.86$, MHC-II $R_S = 0.86$), suggesting that the computational models can effectively guide vaccine design prior to experimental validation.

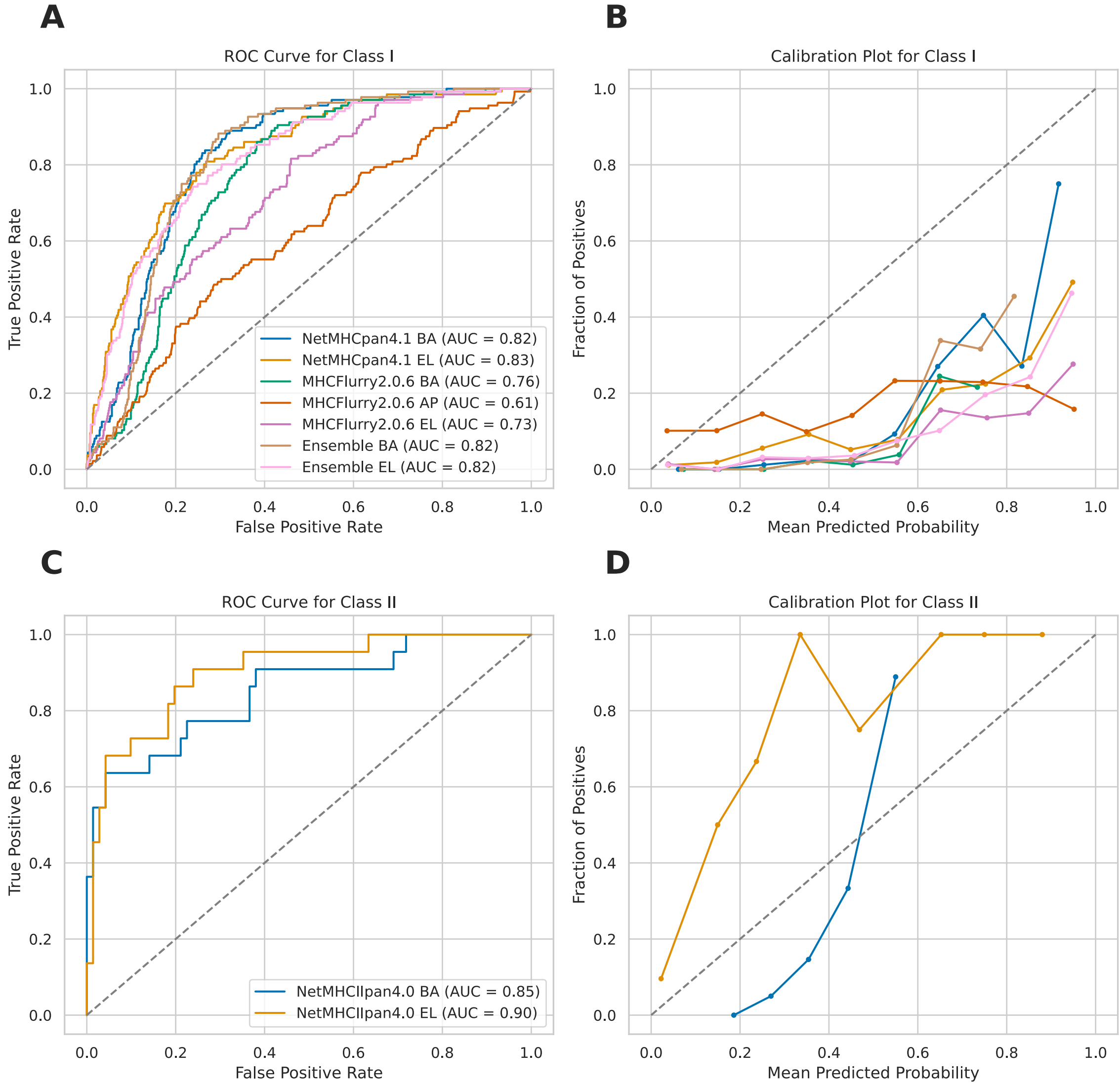

Figure S6. Performance of computational methods for predicting peptide-MHC binding, Related to STAR Methods. Evaluation of computational methods using SARS-CoV-2 peptide stability data across 11 MHC allotypes (5 HLA-A, 1 HLA-B, 4 HLA-C, 1 HLA-DRB1)[9]. The dataset includes 777 unique peptides, 174 of which were binders as determined by the NeoScreen assay. We assessed NetMHCpan v4.1, NetMHCIIpan v4.0[10], and MHCflurry v2.0.6[11], predicting Binding Affinity (BA), Antigen Processing (AP), and Eluted Ligand (EL) scores. (**A**, **C**) Receiver Operating Characteristic (ROC) curves for MHC-I and MHC-II, respectively, showing the trade-off between true positive rate (TPR) and false positive rate (FPR). The area under the ROC curve (AUROC) indicates model performance, with higher values representing better discrimination between binders and non-binders. (**B**, **D**) Calibration plots for MHC-I and MHC-II, respectively, assessing the reliability of predicted probabilities. Perfect calibration aligns with the diagonal dashed line. The NetMHCpan EL score outperformed other methods with AUROC of 0.83 (MHC-I) and 0.90 (MHC-II), and showed good calibration, supporting its use as the default criterion for predicted binding in our study.

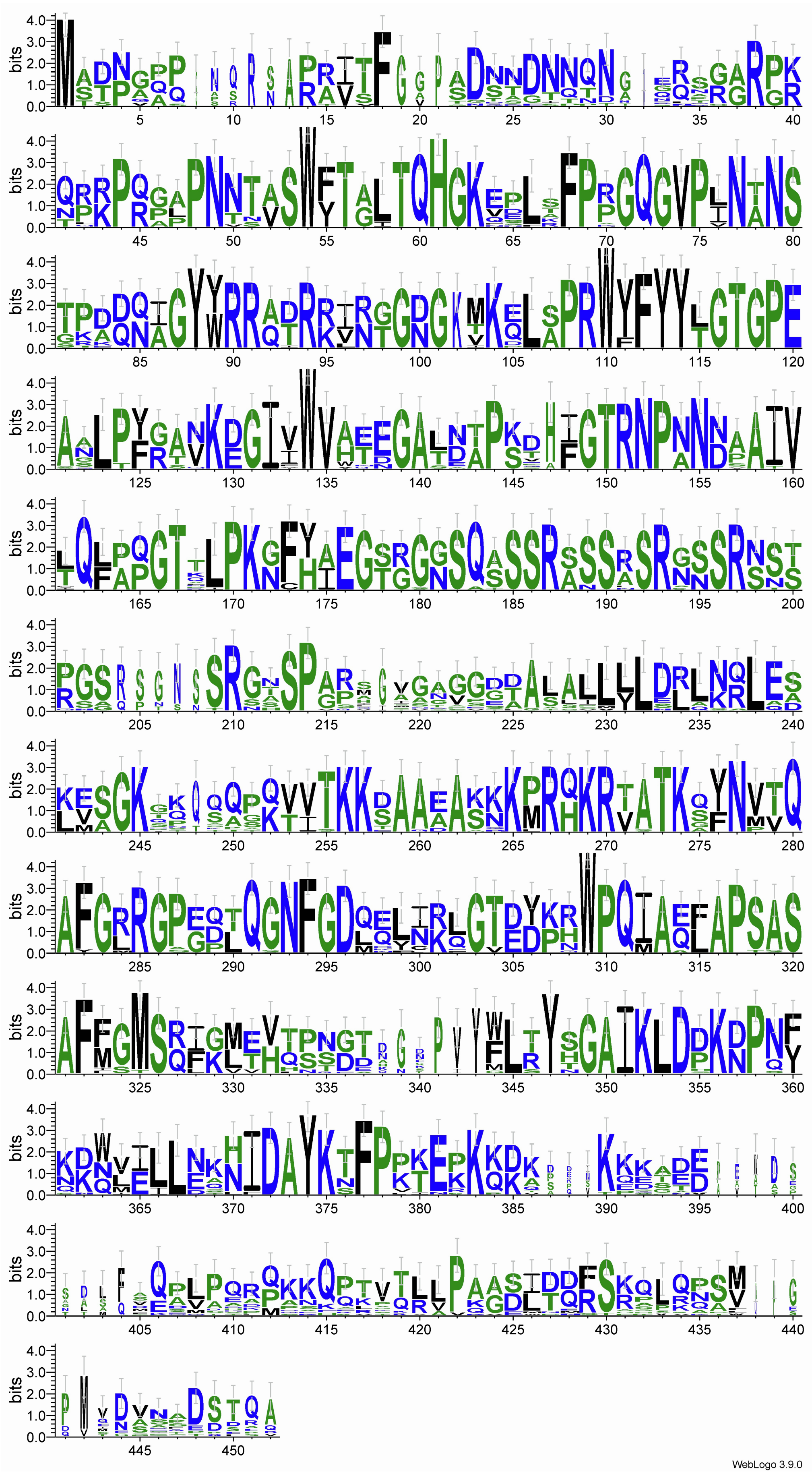

Figure S7. Weblogo of the Nucleoprotein sequences used for antigen design, Related to STAR Methods. The Weblogo was generated using the WebLogo tool[12] from a multiple sequence alignment of the 19 Nucleoprotein sequences used as the target for antigen design. The height of each letter represents the relative frequency of that amino acid at that position, while the overall height of the stack indicates the degree of conservation at that position.

Table S1. Comparison of the expected number of displayed peptides ($\mathbb{E}$[#DPs]) for different antigen designs and ancestries/continents using the dbMHC and AFND databases, Related to Figure 4. The Spectravax antigen was designed and evaluated using haplotype frequencies originally from the dbMHC database. To validate the robustness of the results, an independent dataset of allele frequencies from the Allele Frequency Net Database (AFND) was used to evaluate the $\mathbb{E}$[#DPs] for 95 countries with available data. This table shows a comparison of the $\mathbb{E}$[#DPs] for the antigen design and ancestries/continents using the two databases. The results computed using the two databases are similar, with consistent rankings of the $\mathbb{E}$[#DPs] for all ancestries and antigens, supporting the reliability of the results obtained using the dbMHC database. However, the $\mathbb{E}$[#DPs] computed using AFND are consistently higher than those computed using dbMHC. This discrepancy may be due to the different methods used, as the AFND calculations are based on diploid frequencies, whereas dbMHC calculations are based on haplotype frequencies.

**Antigen**	**Ancestry**	$\mathbb{E}$[**#DPs**] ↑	
		**dbMHC**	**AFND**
Spectravax N	Asian	11.9	14.4
	African	11.8	13.6
	European	10.6	13.4

Table S2. MERS-CoV Nucleocapsid (MN) peptide pool that elicited ELISpot responses, Related to Figure 5. The table lists the peptides, their core sequences, predicted eluted ligand (EL) scores, and percentile ranks. Predictions were made using NetMHCIIpan v4.0[10] for the C57BL/6J mice MHC-II allele H2-IAb. A higher EL Score (↑) and lower EL Rank (↓) indicates a higher likelihood of presentation.

**Name**	**Peptide**	**Core**	**EL Score (↑)**	**EL Rank (↓)**
MN11	VPLNANSTPAQNAGY	ANSTPAQNA	0.1421	6.62
MN12	GNGIKQLAPRWYFYY	IKQLAPRWY	0.0008	85.91
MN13	KQLAPRWYFYYTGTG	WYFYYTGTG	0.0007	87.69
MN14	PRWYFYYTGTGPEAA	YTGTGPEAA	0.4961	1.34
MN15	FYYTGTGPEAALPFR	YTGTGPEAA	0.6110	0.81

Table S3. Datasets used to evaluate Spectravax, Related to STAR Methods. The following antigens were selected from within the *Orthocoronavirinae* subfamily (referred to as *Coronavirinae* for brevity): nucleoprotein (N), spike (S) and non-structural protein 12 (nsp12). RBD refers to the receptor-binding domain of the spike protein. Influenza A virus (within the *Alphainfluenzavirus influenzae* species) antigens include: hemagglutinin (H3), neuraminidase (N1) and matrix protein 1 (M1). To design a human-specific H3N2 vaccine, only sequences from the human host were downloaded. All numbers refer to the processed datasets.

**Dataset ID**	**Phylogenetic Clade(s)**	**Taxid(s)**	**Protein**	**Number of Sequences**	**Median Sequence Length**	**Number of Unique K-mers**
Coronavirinae nsp12	*Coronavirinae*	2501931	nsp12	26	568	15,952
Sarbeco-Merbeco N	*Sarbecovirus*, *Merbecovirus*	2509511, 2509494	N	19	419	9,459
Sarbecovirus S RBD	*Sarbecovirus*	2509511	S RBD	36	196	3,164
Merbecovirus S RBD	*Merbecovirus*	2509494	S RBD	17	235	5,702
Influenza A M1	Influenza A	11320	M1	227	251	2,747
H3N2 H3	H3N2	119210	H3	689	566	5,934
HxN1 N1	H1N1, H2N1, H3N1, H5N1, H6N1, H7N1, H9N1, H10N1	114727, 114730, 157802, 102793, 119212, 119216, 147762, 352769	N1	139	469	8,646

Table S4. Default parameters used in the Spectravax pipeline, Related to STAR Methods.

Parameter (Symbol)	Description	Default Value
1. Sequence Preprocessing		
`seq_identity`	Threshold for sequence identity for clustering	0.95
2. K-merisation		
`k`	List of k-mer lengths for peptide segmentation	(9, 15)
3. K-mer Filtering		
`conservation_threshold`	Minimum pathogen coverage threshold for k-mers	0.01
4. K-mer Scoring		
`score`	Scoring method for vaccine design	'cov'
`scoring_method`	Method for scoring peptides in coverage calculation	'multiplicative'
`equalise_clades`	Adjust scores by the number of sequences in each clade	False
`n_clusters`	Number of clusters to use for clade equalisation	None
$w_{\texttt{mhc1}}$	Weight for MHC-I host coverage	1
$w_{\texttt{mhc2}}$	Weight for MHC-II host coverage	1
`n_target`	Threshold for filtering peptide hits when computing host coverage	1
`expected_coverage`	Use expected host coverage	True
`binding_criteria`	Criteria used for peptide-MHC binding predictions	'EL-score'
`peptide_chunk_size`	Size of peptide batches	500
`n_threads`	Number of threads for NetMHCpan parallel processing	4
`affinity_predictors`	MHC-I binding prediction tool(s)	['netmhcpan']
`netmhcpan_cmd`	Command for running NetMHCpan	'netMHCpan'
`netmhcIIpan_cmd`	Command for running NetMHCIIpan	'netMHCIIpan'
5. Graph Construction		
`decycle`	Remove cycles from epitope graph	True

References

1. Katoh, K., and Standley, D.M. (2013). MAFFT Multiple Sequence Alignment Software Version 7: Improvements in Performance and Usability. Molecular Biology and Evolution *30*, 772–780. URL: https://www.ncbi.nlm.nih.gov/pmc/articles/PMC3603318/. doi: 10.1093/molbev/mst010.

2. Casasanta, M.A., Jonaid, G.M., Kaylor, L., Luqiu, W.Y., DiCecco, L.A., Solares, M.J., Berry, S., Dearnaley, W.J., and Kelly, D.F. (2023). RETRACTED: Structural Insights of the SARS-CoV-2 Nucleocapsid Protein: Implications for the Inner-workings of Rapid Antigen Tests. Microscopy and Microanalysis *29*, 649–657. URL: https://doi.org/10.1093/micmic/ozac036. doi: 10.1093/micmic/ozac036.

3. Mirdita, M., Schütze, K., Moriwaki, Y., Heo, L., Ovchinnikov, S., and Steinegger, M. (2022). ColabFold: making protein folding accessible to all. Nature Methods *19*, 679–682. URL: https://www.nature.com/articles/s41592-022-01488-1. doi: 10.1038/s41592-022-01488-1. Publisher: Nature Publishing Group.

4. Jumper, J., Evans, R., Pritzel, A., Green, T., Figurnov, M., Ronneberger, O., Tunyasuvunakool, K., Bates, R., Žídek, A., Potapenko, A., Bridgland, A., Meyer, C., Kohl, S.A.A., Ballard, A.J., Cowie, A., Romera-Paredes, B., Nikolov, S., Jain, R., Adler, J., Back, T., Petersen, S., Reiman, D., Clancy, E., Zielinski, M., Steinegger, M., Pacholska, M., Berghammer, T., Bodenstein, S., Silver, D., Vinyals, O., Senior, A.W., Kavukcuoglu, K., Kohli, P., and Hassabis, D. (2021). Highly accurate protein structure prediction with AlphaFold. Nature *596*, 583–589. URL: https://www.nature.com/articles/s41586-021-03819-2. doi: 10.1038/s41586-021-03819-2. Publisher: Nature Publishing Group.

5. Minh, B.Q., Schmidt, H.A., Chernomor, O., Schrempf, D., Woodhams, M.D., von Haeseler, A., and Lanfear, R. (2020). IQ-TREE 2: New Models and Efficient Methods for Phylogenetic Inference in the Genomic Era. Molecular Biology and Evolution *37*, 1530–1534. URL: https://doi.org/10.1093/molbev/msaa015. doi: 10.1093/molbev/msaa015.

6. Le, S.Q., and Gascuel, O. (2008). An Improved General Amino Acid Replacement Matrix. Molecular Biology and Evolution *25*, 1307–1320. URL: https://doi.org/10.1093/molbev/msn067. doi: 10.1093/molbev/msn067.

7. Letunic, I., and Bork, P. (2021). Interactive Tree Of Life (iTOL) v5: an online tool for phylogenetic tree display and annotation. Nucleic Acids Research *49*, W293–W296. URL: https://doi.org/10.1093/nar/gkab301. doi: 10.1093/nar/gkab301.

8. Cock, P.J.A., Antao, T., Chang, J.T., Chapman, B.A., Cox, C.J., Dalke, A., Friedberg, I., Hamelryck, T., Kauff, F., Wilczynski, B., and de Hoon, M.J.L. (2009). Biopython: freely available Python tools for computational molecular biology and bioinformatics. Bioinformatics *25*, 1422–1423. URL: https://doi.org/10.1093/bioinformatics/btp163. doi: 10.1093/bioinformatics/btp163.

9. Prachar, M., Justesen, S., Steen-Jensen, D.B., Thorgrimsen, S., Jurgons, E., Winther, O., and Bagger, F.O. (2020). Identification and validation of 174 COVID-19 vaccine candidate epitopes reveals low performance of common epitope prediction tools. Scientific Reports *10*, 20465. URL: https://www.nature.com/articles/s41598-020-77466-4. doi: 10.1038/s41598-020-77466-4. Number: 1 Publisher: Nature Publishing Group.

10. Reynisson, B., Alvarez, B., Paul, S., Peters, B., and Nielsen, M. (2020). NetMHCpan-4.1 and NetMHCIIpan-4.0: improved predictions of MHC antigen presentation by concurrent motif deconvolution and integration of MS MHC eluted ligand data. Nucleic Acids Research *48*, W449–W454. URL: https://doi.org/10.1093/nar/gkaa379. doi: 10.1093/nar/gkaa379.

11. O'Donnell, T.J., Rubinsteyn, A., and Laserson, U. (2020). MHCflurry 2.0: Improved Pan-Allele Prediction of MHC Class I-Presented Peptides by Incorporating Antigen Processing. Cell Systems *11*, 42–48.e7. URL: https://www.sciencedirect.com/science/article/pii/S2405471220302398. doi: 10.1016/j.cels.2020.06.010.

12. Crooks, G.E., Hon, G., Chandonia, J.M., and Brenner, S.E. (). WebLogo: a sequence logo generator. *14*, 1188–1190. doi: 10.1101/gr.849004.
